# Using an experiment among clinical experts to determine the cost and clinical impact of rapid whole exome sequencing in acute pediatric settings

**DOI:** 10.3389/fped.2023.1204853

**Published:** 2023-07-03

**Authors:** Nattiya Kapol, Wuttichart Kamolvisit, Lalida Kongkiattikul, Evan Huang-Ku, Namfon Sribundit, Surasit Lochid-Amnuay, Nathapol Samprasit, Thamonwan Dulsamphan, Parntip Juntama, Chotika Suwanpanich, Ponghathai Boonsimma, Vorasuk Shotelersuk, Yot Teerawattananon

**Affiliations:** ^1^ Faculty of Pharmacy, Silpakorn University, Nakhon Pathom, Thailand; ^2^ Center of Excellence for Medical Genomics, Faculty of Medicine, Chulalongkorn University, Bangkok, Thailand; ^3^ Excellence Center in Genomics and Precision Medicine, King Chulalongkorn Memorial Hospital, Thai Red Cross Society, Bangkok, Thailand; ^4^ Pediatric Critical Care, Department of Pediatrics, Faculty of Medicine, Chulalongkorn University, Bangkok, Thailand; ^5^ Dalla Lana School of Public Health, University of Toronto, Toronto, Canada; ^6^ Department of Pharmacy, Bangpakok 8 Hospital, Bangkok, Thailand; ^7^ Ministry of Public Health, Health Intervention and Technology Assessment Program (HITAP), Nonthaburi, Thailand; ^8^ Saw Swee Hock School of Public Health, National University of Singapore, Singapore, Singapore

**Keywords:** rapid whole-exome sequencing, cost-effectiveness, next-generation sequencing, low- and middle-income countries, acute pediatrics

## Abstract

**Objective:**

Evaluate the cost and clinical impacts of rapid whole-exome sequencing (rWES) for managing pediatric patients with unknown etiologies of critical illnesses through an expert elicitation experiment.

**Method:**

Physicians in the intervention group (*n* = 10) could order rWES to complete three real-world case studies, while physicians in the control group (*n* = 8) could not. Costs and health outcomes between and within groups were compared.

**Results:**

The cost incurred in the intervention group was consistently higher than the control by 60,000–70,000 THB. Fewer other investigation costs were incurred when rWES could provide a diagnosis. Less cost was incurred when an rWES that could lead to a change in management was ordered earlier. Diagnostic accuracy and the quality of non-pharmaceutical interventions were superior when rWES was available.

**Conclusion:**

In acute pediatric settings, rWES offered clinical benefits at the average cost of 60,000–70,000 THB. Whether this test is cost-effective warrants further investigations. Several challenges, including cost and ethical concerns for assessing high-cost technology for rare diseases in resource-limited settings, were potentially overcome by our study design using expert elicitation methods.

## Introduction

Genetic disorders in children contribute to acute hospitalizations and mortality (1–3). Early diagnosis is critical for better outcomes but difficult to achieve due to atypical presentations for thousands of potential genetic diseases (4). Next-generation sequencing (NGS) is a precision medicine that can scan a large amount of DNA rapidly for a diagnosis, which has demonstrated reduced morbidity, mortality, length of stay, and unnecessary procedures in hospitalized children (5–7). Unlike traditional genetic sequencing, NGS is more advantageous due to its shorter turnaround time, which is critical in acute settings (8). In certain instances, an earlier diagnosis also helps to make palliative care decisions (5, 6). In a Thai study, rapid whole exome sequencing (rWES), a type of NGS, led to a diagnosis in 46% of patients with *unknown etiologies of critical illnesses,* which resulted in a change in clinical management and improved outcomes (9). While NGS may have tremendous benefits and is becoming increasingly available in higher-income countries due to declined costs, it has not yet gained wide-scale implementation in lower-income countries (10). To our knowledge, there are only a couple of economic studies (11, 12) on NGS in Thailand and none about using rWES in pediatric patients with unknown etiologies of critical illnesses. Therefore, this study aims to generate cost data and assess clinical outcomes of pediatric patients with unknown etiologies of critical illnesses when rWES is available to Thai physicians.

Collecting clinical trial data on pediatric patients with unknown etiologies of critical illnesses for an economic study is challenging – getting an adequate sample size is near impossible for this rare condition, and withholding a potentially life-saving intervention is highly unethical. As such, the research team employed an expert elicitation design to acquire the necessary data bypassing the aforementioned challenges. With the results of this study, the research team aims to conduct a follow-up cost-effectiveness study to inform population health decisions, including reimbursing rWES in Thailand's universal health benefits package.

## Methods

### Study design

The experiment randomly assigned clinical experts to the intervention group or the control group to undergo expert elicitation on three real-world patient case studies. Experts assigned to the intervention group could order rWES tests for all three case studies when performing the elicitation task, while experts in the control group could not. For each case study, experts were asked to investigate and provide treatment at each of the three visits. At the third visit, experts were asked to diagnose the patient, predict the prognosis at one-year post-discharge, and write a discharge plan, including non-pharmaceutical interventions. A detailed illustration of the expert elicitation experiment, the case studies, order and treatment forms, and laboratory results are available in the Supplementary Material. The development of the study design closely followed recommendations from the Reference Case Methods for Expert Elicitation in Health Care Decision Making with appropriate adaptations (13).

### Sample selection and sampling

A survey distributed to the Royal College of Physicians in Thailand was used to recruit as many experts as possible for the full range of expert opinions. Inclusion criteria were: (1) currently practicing in pediatric critical care medicine or related critical care, (2) willingness to participate in the in-person experiment in Bangkok. Exclusion criteria were: (1) physicians without formal training in pediatrics and (2) physicians who treated the patients in the three real-world case studies.

Ten and eight experts were assigned to the intervention and control groups through stratified random sampling. The stratifications were gender, years of experience (under or over ten years), location of work (intra- or extra-Bangkok), and work setting (private or public). The intervention group had two more experts than the control to generate additional variations in response in the intervention group. The sample size in this study was adequate as per the Reference Case Methods for Expert Elicitation in Health Care Decision Making, which recommended at least five experts to balance different viewpoints in conducting expert elicitations (13).

### Case selection

Cases were selected from a 2021 study that reported on 54 patients admitted to ICUs or inpatient wards with seriously ill conditions without obvious causes recruited from 11 tertiary hospitals in Thailand between 2018 and 2020 (9). Adults (*n* = 7) were excluded from the case selection because they constituted a smaller proportion of the cases and were treated by physicians without pediatric training. Thus, focusing the study on physicians treating children was more feasible. The remaining 47 pediatric cases were assigned into three strata based on their real-world rWES results and outcomes: (1) rWES provided a diagnosis and caused a change in clinical management (*n* = 20), (2) rWES provided a diagnosis but did not result in a change in clinical management (*n* = 1), (3) rWES did not provide a diagnosis and did not change the clinical management (*n* = 26). One sample was randomly selected for Stratum 1 and 3.

Due to jurisdictional restrictions in accessing the clinical data of the patient in Strata 2, the research team chose a patient from Strata 1 that fits more closely with the definition of Strata 2. For this chosen case, an rWES test provided a diagnosis that could inform the chronic management but not an immediate change in management. A detailed summary of the three cases is available in the Supplementary Material.

### Materials and data collection

The materials for data collection were adapted from forms used within hospitals to enhance the simulation experience and minimize procedure-based errors. The materials are available upon request.

This experiment was piloted once with experts not recruited for the study to inform the final protocol. For example, additional time was allotted for the experiment to allow for more meaningful investigations.

Two research coordinators with extensive experience managing pediatric patients in acute settings were recruited to oversee the elicitation task for each group. They were responsible for providing instructions, timing each case study, and responding to emerging problems. Pharmacists, researchers, and research assistants were recruited as facilitators to help individual experts by providing the materials required for the task and answering procedural-related questions - see the procedures illustrated in Supplementary Material. Both research coordinators and facilitators received prior training in their respective roles. Lastly, experts were assigned a code to de-identify elicitation outcomes. All experts were briefed on this study's objectives, procedures and expected benefits and provided written consent to participate before the experiment.

### Outcomes

The primary outcome was the cost incurred per physician, calculated from the laboratory tests and treatments ordered. At each visit, experts selected options from a pre-determined list of investigatory laboratory tests and wrote a treatment plan. Laboratory test results, including rWES results, were provided at the next visit. On the order form, physicians were informed that the turnaround time for rWES was three days. Ordering tests not on the pre-determined list was permitted. However, the ordering physicians would be told later that the results were unavailable. These steps were repeated on the second and final visit. At the last visit, physicians were also prompted to write about discharge medications. Two weeks' worth of medication was used if the physicians did not specify the duration of the discharge medication. The cost was calculated by the quantity of material used, deduced by two clinical experts, multiplied by the unit cost retrieved from three sources: the Drug and Medical Supply Information Centre, Ministry of Public Health (drug cost), Chulalongkorn University (lab and procedure cost), and Health Intervention Technology Assessment Program (HITAP) for drug and procedure costs, adjusted to the year 2022. Furthermore, the average costs incurred by physicians that ordered rWES in visit 1 compared to visits 2 and 3 combined were also calculated to investigate the relationships between costs and the temporality of rWES orders.

Secondary outcomes were the clinical impact represented by the number of physicians in each group that made a correct diagnosis and prognosis and ordered complete and accurate non-pharmaceutical interventions. At the final visit, experts were asked to diagnose and predict the one-year post-discharge prognosis by selecting one option: (1) alive with controlled symptoms, (2) alive with uncontrolled symptoms, (3) deceased, and (4) unsure. Clinical experts, as part of the research team, determined a correct diagnosis based on specificity and accuracy, i.e., a definite diagnosis was deemed correct, but a diagnosis of the disease group may be incorrect. See Supplemental Material for examples of diagnostic correctness based on specificity. The prognosis is evaluated against the real-world case outcomes: Cases 1 and 3 were alive without complication, and Case 2 was deceased. Lastly, non-pharmaceutical interventions were measured through physicians' discharge plans written on a blank page at the last visit. Based on the response, the research team determined whether the physicians had completely and correctly planned for (1) providing family planning advice, (2) providing behavioural modification advice, and (3) issuing a medical ID letter. A medical ID letter is a piece of paper with the patient's medical information that should be carried by the patient to help the healthcare team (paramedics or the emergency department) respond to an emergency.

### Data analysis

Physician gender, affiliated hospital, and region were compared using *χ*^2^ tests, and years of experience were compared using the Mann–Whitney *U* test. The direct medical cost between groups for each case study was compared using the Independent-Samples Mann–Whitney *U* test. The average costs incurred by physicians that ordered rWES were compared using the Independent-Sample Mann–Whitney *U* test. The difference between groups for correct diagnosis, prognosis, and NPI were compared using Fisher's exact test. Statistical significance was set at *p*-value <0.05. All statistical analyses were conducted using SPSS Statistical Software.

### Ethics review

Ethics approval was acquired through Silpakorn University's Human Research Ethics Review Board which determined compliance with ethical principles.

## Results

Eighteen participants were recruited for the study. Most were female (*n* = 11) working in university hospitals (*n* = 7) in the Bangkok region (*n* = 12). The years of experience ranged from two to twenty years. The characteristics of physicians in the two groups were not significantly different at *p*-value <0.05. Detailed information on the physicians' characteristics is listed in Table 1.

**Table 1:** Physician characteristics between the control and intervention groups undergoing the expert elicitation task (Thailand, 2022).

Characteristics	Control (*n* = 8)	Intervention (*n* = 10)	*p*-value
	*n* (%)	*n* (%)	
Gender			0.648
Male	3 (37.5)	4 (40.0)	
Female	5 (62.5)	6 (60.0)	
Affiliation Hospital			0.850
University hospital	3 (37.5)	4 (40.0)	
Regional hospital	1 (12.5)	2 (20.0)	
Specialized hospital	2 (25.0)	3 (30.0)	
General hospital	2 (12.5)	1 (10.0)	
Province			0.563
Bangkok	5 (62.5)	7 (70.0)	
Other provinces	3 (37.5)	3 (30.0)	
Working Experience (year)			0.573
Min	2	3	
Max	20	20	
Average	7.75	8.5	
SD	6.32	5.36	

Min, minimum; Max, maximum; SD, standard deviation.

The direct medical cost incurred per physician between the two groups was summarized in Table 2 by the cost of investigation, cost of treatment, and total cost. For Case 1, although the cost was higher in the intervention group, it was not significantly different from the control group, tested by the Mann–Whitney *U* test. For Cases 2 and 3, the cost of investigation and the total cost were higher in the intervention group, with statistical significance using the Mann–Whitney test. The absolute cost difference between the control and intervention groups was summarized in Table 3.

**Table 2:** Total cost, investigation cost, and treatment cost, in Thai baht, calculated with and without the cost of rapid exome sequencing, incurred between the control and the intervention groups (Thailand, 2022).

	Case 1			Case 2			Case 3		
	Control (95% CI)	Intervention (95% CI)	*p*-value	Control (95% CI)	Intervention (95% CI)	*p*-value	Control (95% CI)	Intervention (95% CI)	*p*-value
Total cost with rWES (x1,000 THB)									
Cost of investigation	79.9 (44.3–115.6)	121.3 (91.1–151.4)	0.122	80.9 (56.6–105.3)	143.8 (111.8–175.8)	0.004*	12.6 (4.8–20.5)	83.8 (48.7–118.9)	0.003*
Cost of treatment	69.8 (46.2–93.3)	88.8 (53.2–124.4)	0.460	14.8 (11.2–18.5)	13.9 (11.4–16.4)	0.696	74.1 (62.1–86.1)	76.3 (65.1–87.4)	0.829
Total cost	149.7 (101.1–198.3)	210.0 (161.3–258.7)	0.146	95.7 (71.0–120.5)	157.7 (125.9–189.5)	0.003*	86.7 (70.8–102.7)	160.1 (117.1–203.1)	0.009*
Total cost without rWES (x1,000 THB)									
Cost of investigation	79.9 (44.3–115.6)	57.3 (32.2–82.3)	0.237	80.9 (56.6–105.3)	71.8 (44.6–99.0)	0.460	12.6 (4.8–20.5)	35.8 (24.4–47.3)	0.009*
Cost of treatment	69.8 (46.2–93.3)	88.8 (53.2–124.4)	0.460	14.8 (11.2–18.5)	13.9 (11.4–16.4)	0.696	74.1 (62.1–86.1)	76.3 (65.1–87.4)	0.829
Total cost	149.7 (101.1–198.3)	146.0 (95.3–196.8)	0.965	95.7 (71.0–120.5)	85.7 (58.1–113.2)	0.315	86.7 (70.8–102.7)	112.1 (94.9–129.3)	0.021*

rWES, rapid whole-exome sequencing; CI, confidence interval; THB, Thai baht.

*Refers to a *p*-value lower than 0.05.

**Table 3:** The absolute cost differences between groups with and without the cost of rapid whole-exome sequencing, in thousands of Thai baht (Thailand, 2022).

	Case 1	Case 2	Case 3
Absolute cost difference with rWES cost in the intervention group (x1,000 THB) (95% CI)			
Cost of investigation	41.3 (35.8–46.8)	62.9 (55.2–70.5)	71.2 (43.9–98.5)
Cost of treatment	19.0 (6.9–31.1)	0.9 (0.2–2.0)	2.2 (1.3–3.1)
Total cost	60.3 (60.2–60.4)	61.9 (54.9–69.0)	73.4 (46.2–100.5)
Absolute cost difference without rWES cost in the intervention group (x1,000 THB) (95% CI)			
Cost of investigation	22.7 (12.0–33.3)	9.1 (6.3–12.0)	23.2 (19.6–26.8)
Cost of treatment	19.0 (6.9–31.1)	0.9 (0.2–2.0)	2.2 (1.3–3.1)
Total cost	3.7 (1.5–5.8)	10.1 (7.2. 12.9)	25.4 (24.0–26.7)

rWES, rapid whole-exome sequencing; CI, confidence interval; THB, Thai baht.

The comparison between the average investigation, treatment, and total cost incurred by physicians who ordered rWES in visit 1 and visits 2 and 3 combined was summarized in Table 4. For Case 1, the cost of investigation incurred by physicians that ordered rWES in visit 1 was lower than when physicians ordered rWES in visits 2 and 3 combined, and the difference was significant.

**Table 4:** The cost comparison between physicians that ordered rapid-whole exome sequencing in visit 1 vs. visits 2 and 3 combined, in thousands of Thai Baht (Thailand, 2022).

	Case 1				Case 2				Case 3			
	*n*	Cost of investigation (SD)	Cost of treatment (SD)	Total cost (SD)	*n*	Cost of investigation (SD)	Cost of treatment (SD)	Total cost (SD)	*n*	Cost of investigation (SD)	Cost of treatment (SD)	Total cost (SD)
Visit 1	3	104.6 (1.2)	64.3 (14.3)	169.0 (13.1)	3	127.9 (2.3)	12.1 (0.8)	140.0 (2.7)	0	–	–	–
Visits 2 and 3, combined	5	150.3 (40.1)	92.7 (54.8)	242.9 (77.8)	6	163.0 (45.8)	14.2 (4.2)	177.2 (45.7)	6	120.0 (15.4)	84.6 (13.9)	204.6 (17.7)
*p*-value		0.036*	0.571	0.143		0.381	0.714	0.167		–	–	–

rWES, rapid whole-exome sequencing;

n, Number of physicians who ordered an rWES test; SD, standard deviation.

*Refers to a *p*-value lower than 0.05.

Results for diagnostic correctness, prognosis accuracy, and NPI were summarized in Table 5. More physicians in the intervention group made a correct diagnosis for Cases 1 and 2 with statistical significance confirmed by Fisher's exact test. The prognosis accuracy was not significantly different between groups. Only physicians in the intervention group provided NPI in discharge planning for Cases 1 and 2. However, the difference is only significant in Case 1 for providing behavioral modification advice (*p* = 0.036) and issuing medical ID letters (*p* = 0.013).

**Table 5:** Comparison of diagnostic correctness, prognostic accuracy, and non-pharmaceutical interventions represented by advice for family planning, advice for behavioural modification, and issuing medical identification letters (Thailand, 2022).

	Case 1			Case 2			Case 3		
	Control	Intervention	*p*-value	Control	Intervention	*p*-value	Control	Intervention	*p*-value
Diagnostic score									
Correct	0	6	0.013*	3	10	0.007*	5	2	0.145
Incorrect	8	4		5	0		3	8	
Prognosis score									
Correct	5	6	1.00	4	2	0.321	7	4	0.066
Incorrect	3	4		4	8		1	6	
Advice for family planning									
Complete	0	4	0.092	0	2	0.477	8	10	-
Incomplete	8	6		8	8		0	0	
Advice for behavioral modification									
Complete	0	5	0.036*	0	3	0.216	2	0	0.183
Incomplete	8	5		8	7		6	10	
Issuing a medical ID letter									
Complete	0	6	0.013*	0	3	0.216	0	1	1.000
Incomplete	8	4		8	7		8	9	

ID, identification.

*Refers to a *p*-value lower than 0.05.

## Discussion

The cost of rWES (trio-rWES) in Thailand at the time of writing was 80,000 THB (approximately 2,250 USD based on the exchange rate in 2022), comparable to the cost in high-income settings and relatively high in proportion to the costs incurred in the intervention arm: rWES constituted 38%, 51%, 50% of the total cost in cases 1, 2, and 3. The high cost of rWES may explain why the investigation and total cost in the intervention group were consistently higher than the control, as seen in Table 2. Should rWES cost reduce in the future, we believe the availability of this technology in Thailand will not become an economic burden to the healthcare system. Currently, the Thai government is investigating personalized medicines for inclusion in universal health coverage (UHC), i.e., a parallel study is undertaken to assess the cost of genetic testing for intractable epilepsy. As genetic testing, such as rWES, becomes increasingly available, the cost will reduce through two mechanisms. First, through economies of scale, where a larger test quantity will reduce the significance of its fixed costs, reducing the cost per test (14, 15). Economies of scale can also be seen when payers purchase larger quantities of the material at a discounted price (14). Second, when universally covered indications for exome sequencing and other genetic tests expand, we may observe economies of scope that reduce the cost per test by synergistically sharing input, such as facilities and human resources to process tests unrelated to idiopathic acute symptoms. Economies of scale and scope in the health service context have been documented extensively in the literature (14, 15).

When dividing the total cost into investigation and treatment costs, we also noted that physicians in the intervention group incurred fewer costs from other investigations in Cases 1 and 2, although only significant in Case 1, as seen in Table 2. Given that rWES can provide a definite diagnosis in Cases 1 and 2, physicians in the intervention group likely did not need to order as many other investigations. Not surprisingly, the cost of other investigations was not lower in the intervention group for Case 3 since rWES could not provide a definite diagnosis.

When comparing the cost incurred by physicians that ordered rWES, it was noted that those in visit 1, compared to those in visits 2 and 3 combined, incurred less investigation and treatment cost for Case 1, although only significant for the cost of investigation (Table 4). We hypothesize that knowing the rWES results earlier allowed physicians to avoid subsequent unnecessary investigation and treatment expenditures. Although rWES should not offer immediate clinical benefits for patients in Strata 2 since its results cannot cause a change in management, we hypothesize that rWES may reduce physicians' stress when treating critically ill patients as it can determine a diagnosis. A future study is underway to understand the association between physician stress and access to genetic test options.

Although the availability of the rWES test led to higher spending, it also offered clinical benefits, especially for patients that fall under the Case 1 stratum, where an accurate diagnosis could lead to a change in clinical management. This may be the most common type of patient - of the 47 idiopathic severe illness cases used in case selection, 20 (43%) fall into this stratum (9). An accurate diagnosis may have led to physicians providing helpful NPI in their discharge plans, such as family planning and behavioural modification advice and issuing medical ID letters. These NPIs offer benefits beyond the length of stay during hospitalization (16), as seen in the management of other genetic diseases (17–20). Ultimately, the clinical benefits of rWES are realized at approximately 60,000–70,000 THB. Whether this cost is cost-effective warrants a follow-up study.

Our study revealed clinical benefits such as more accurate diagnosis and NPIs in discharge planning when the rWES test was available, with lower economic consequences the earlier it was ordered. This finding can inform the clinical practice guidelines in that ordering rWES in idiopathic critical illness as a first-line investigation may be warranted for both clinical and economic benefits. Next-generation sequencing has also been recommended as a first-line investigation in patients suspected of genetic diseases to prevent a diagnostic odyssey, where the patient undergoes years of serial testing, incurring costly medical expenses in the hopes of finding a diagnosis (21–23).

Interestingly, physicians in the intervention group used rWES rather appropriately. Cases 1 and 2 were medically complicated to diagnose. As such, 80% and 90% of the physicians in Cases 1 and 2 resorted to ordering rWES. In contrast, Case 3 was less medically complicated and could be diagnosed with immunology profile tests, likely explaining that only 60% of the physicians ordered rWES. Experts in this study were physicians with real-world experience in pediatric critical care medicine. It is reasonable to assume that the cost and clinical outcomes would look very different if physicians without this expertise were recruited for this study.

Our study result is invaluable, conducted in a low-middle-income setting, to inform health policy decisions, such as whether to fund rWES through universal health coverage based on anticipated cost and clinical impact. We consider the research design an ex-ante approach, defined by evaluating the clinical and economic impact of rWES through an expert elicitation using case studies before providing universal coverage for this technology. The use of ex-ante and ex-post evaluations for public health products and policies has been documented in the literature (24–26). The expert elicitation approach is advantageous by avoiding the cost of conducting a trial-based analysis, where follow-ups to patient outcomes over a sufficient time horizon are resource intensive (10), especially in rare conditions and for low-resource settings. Therefore, we recommend that lower-middle-income countries consider using expert elicitations when clinical trials are challenging to conduct. In higher-income settings where rWES is used routinely, an ex-post approach can be used to understand the real-world economic and clinical impact. We encourage researchers to compare ex-ante and ex-post health economic study designs to evaluate the validity of expert elicitations.

The most common expert elicitation approach in economic evaluation is the unblind self-control method. In this approach, experts are provided with the outcome of a test or treatment and predict the cost and outcomes as if they did not know the test results or treatment. However, knowing the outcome in advance can influence expert judgment. Therefore, our study used the blinded self-control method instead. We believe our approach provided a more unbiased result.

There are several limitations to this study. Physicians in real-world settings may have more time, resources, investigation, and treatment options than presented in the experiment, which could affect the accuracy of cost and health outcomes. Although we assumed all physicians put in their best effort to complete the case studies, we could not control the quality of their input. Recruitment of physicians was non-random, and participation was volunteered, which could have added selection bias for physicians wishing to advance access to rWES. The generalizability of the expert opinion result has limits as most physicians in the study practiced in Bangkok, and medical training varies significantly between countries, especially in Southeast Asia. All cases were selected from a cohort of patients in Thailand, which may not represent patient profiles from other countries. In addition, we could not account for how rWES would affect the patient's length of stay in this experiment, the benefits of an earlier discharge resulting from an accurate diagnosis, downstream treatment cost because of a diagnosis, and costs unrelated to health services (10). Lastly, a scenario where a negative rWES test led to a change in management was not accounted for in the study. A negative rWES result could be beneficial in ruling out genetic diseases. Thus, the benefit of rWES could be even greater than that captured by the study.

In conclusion, the availability of rWES did not reduce direct medical costs by avoiding unnecessary investigation and treatment procedures. Our study found that the average cost incurred per physician for managing pediatric patients with unknown etiologies of critical illness was higher by 60,000–70,000 THB when rWES was available. However, when rWES was available, physicians were more likely to diagnose patients with unknown etiologies of critical illness accurately, offer behavioural modification advice, and issue medical ID letters, which could lead to tremendous and positive clinical implications. Moreover, physicians incurred less investigation cost when rWES was ordered earlier in patients that could be diagnosed through an rWES test. Whether the benefits are cost-effective warrants further study. This study adds to the existing knowledge by investigating the implication of rWES on Thai physicians' management of patients and the clinical and cost consequences, using an expert elicitation design that can be replicated in resource-limited settings to inform policymakers' decisions to fund this technology in the universal health package.

## Data Availability

The raw data supporting the conclusions of this article will be made available by the authors, without undue reservation.
