# Supplementary material for: Using an experiment among clinical experts to determine the cost and clinical impact of rapid whole exome sequencing in acute pediatric settings
Source: Front Pediatr. 2023 Jul 3;11:1204853. doi: 10.3389/fped.2023.1204853 (PMC10350589; doi:10.3389/fped.2023.1204853)
Supplement: Supplementary file 1 [file Table1.docx]

# SUPPLEMENTARY MATERIALS

Journal name: Frontiers in Pediatrics

Article title: Using an experiment among clinical experts to determine the cost and clinical impact of rapid whole exome sequencing in acute pediatric settings.

Figure S1. An illustration of the expert elicitation task procedure and interactions between the facilitators and the experts (Thailand, 2022).

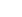

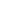

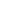


Table S1. The detailed summary of the case studies selected for the expert elicitation tasks, including patient presentation, diagnosis, and prognosis (Thailand, 2022).

| Strata | Definition | Case description |
| --- | --- | --- |
| 1 | rWES revealed a diagnosis and caused a change in management | The patient presented with an alternation of consciousness and severe wide-gap metabolic acidosis. The molecular diagnosis is HMG CoA synthase 2 deficiency. Treatments were hemodialysis and a high concentration of IV glucose to prevent hypoglycemia. The discharge status was alive and well. |
| 2 | rWES revealed a diagnosis but did not result in a change in management | The patient presented with an alternation of consciousness. The molecular diagnosis is ornithine transcarbamylase (OTC) deficiency. This case is classified into the group of “no or minimal change in management” because the clinical manifestations and the findings of severe hyperammonemia were sufficient for the treating physician to give a diagnosis of urea cycle defects. Thus, hemodialysis, restricted proteins, ammonia scavenger treatment were provided before the molecular diagnosis from rWES was made. Unfortunately, the patient’s condition was not stable enough to be referred to the tertiary medical center and the patient died because of prolonged hyperammonemia and severe encephalopathy at the primary hospital. |
| 3 | rWES was unable to give a diagnosis and did not change the management | The patient presented with severe diarrhea and shock. With a positive dihydrorhodamine test, a diagnosis of chronic granulomatous disease (CGD) was given. rWES result was negative. Subsequently, PCR-Restriction Fragment Length Polymorphism (RFLP) revealed that the patient was homozygous for a small deletion in *NCF1*, providing a molecular diagnosis. Treatments included antibiotic prophylaxis and hematopoietic stem cell transplant (HSCT). The patient recovered and could be discharged from the hospital. |

^rWES: rapid-exome sequencing^

Table S2: Examples of diagnosis and their correctness, determined by diagnostic specificity, according to two clinical experts (Thailand, 2022).

| Case # | Diseases | Correct | Examples of diagnosis ordered in increasing specificity |
| --- | --- | --- | --- |
| 1 | Mitochondrial HMG-CoA Synthase Deficiency (HMGCS2) | No | Septic shock |
|  |  | No | Inborn errors of metabolism |
|  |  | No | Mitochondrial disorders |
|  |  | Yes | Mitochondrial HMG-CoA Synthase Deficiency |
| 2 | Ornithine Transcarbamylase deficiency | No | Septic shock |
|  |  | No | Inborn errors of metabolism |
|  |  | Yes | Urea cycle defect |
|  |  | Yes | Ornitine Transcarbamylase Deficiency |
| 3 | Chronic Granulomatous Disease | No | Septic shock |
|  |  | No | Recurrence infection |
|  |  | No | Primary immune deficiency |
|  |  | Yes | Chronic Granulomatous Disease |

Materials used in the experiment for all three cases, including the case studies, laboratory order forms, laboratory results, diagnostic sheets, and treatment order sheets.

Case #1

**Case scenario 1**

**ผู้ป่วยรายนี้มีทั้งหมด 3 evaluations**

evaluation ที่ 1 at first day of admission

evaluation ที่ 2 at day 3 of admission

evaluation ที่ 3 at day 24 of admission (discharge date)

- **1st evaluation at first day of admission**
- ทารกเพศหญิงอายุ 9 เดือน
- อาเจียนมาก 2 วัน ก่อนมา ร.พ.
- 2 วันก่อน อาเจียนออกมาเป็นนมปนน้ำลายครั้งละประมาณ 1 แก้ว มากกว่า 10 ครั้ง กินนมได้ลดลง ไม่มีไข้ ไม่มีท้องเสียถ่ายเหลว ไม่มีอาการอื่น มารดาพาไปคลินิก แพทย์วินิจฉัยว่าเป็นไข้หวัด ได้ยากลับบ้านเป็น ยาลดไข้ ยาแก้อาเจียน
- 1 วันก่อนมาโรงพยาบาล ผู้ป่วยไม่มีอาเจียนแต่ไม่มีปัสสาวะเลยทั้งวัน เริ่มซึม ปลุกตื่นยาก ไม่มีอาการอื่น มารดาจึงพามาโรงพยาบาล
- ระหว่างรอตรวจหน้าห้องฉุกเฉิน มีเกร็งกระตุกทั้งตัว ตาลอย คอบิด เป็นอยู่ประมาณ 30 วินาที มีการรักษาโดยให้ Diazepam 0.3 mg/kg/dose IV
- ทางห้องฉุกเฉินประเมินว่าผู้ป่วยซึมมากจึงมีการใส่ ET Tube No. 4
- ปฏิเสธโรคประจำตัว
- ปฏิเสธประวัติแพ้ยา และ/หรือ อาหาร
- ปฏิเสธ drug/toxic substance ingestion
- ปฏิเสธประวัติอุบัติเหตุ
- ประวัติคลอด
- Term C/S due to previous c/s ที่ GA 38 weeks BW 2,584g (P25-50th) AGA APGAR 9,10 Head circumference 33 cm (P25-50th) มารดาฝากครรภ์ปกติ serology negative all ไม่มีไข้/น้ำเดินก่อนคลอด
- กินแต่นมแม่จนถึงอายุ 7 เดือน
- เริ่มให้นมผงตั้งแต่อายุ 7 เดือน และอาหารตามวัย 1-2 มื้อ
- วัคซีนครบตาม EPI + Hib3
- พัฒนาการ
- Gross motor: นั่งได้เองไม่ต้องจับ, เกาะยืนได้
- Fine motor: คว้าของด้วยฝ่ามือได้
- Language: พูดหม่ำๆได้ ยิ้มตอบได้ บ๊ายบายได้
- Social: หันตามเสียงเรียกได้ กลัวคนแปลกหน้า

**Pedigree of three generations**

- ปฏิเสธการแต่งงานในเครือญาติ
- ปฏิเสธคนในครอบครัว เสียชีวิตไม่ทราบสาเหตุ ชัก หรือ พัฒนาการล่าช้า

**Physical examination**

- Measure: BW 8 kg (P50), Lt 69 cm (P25-50), HC 45 cm (P75)
- V/S: BT 38.6 C, PR 158 bpm[Full], RR74/min, BP 107/62 mmHg, SpO2 100%
- GA: A Thai girl, on ETT, Comatose
- Skin: No rash, no petichiae
- HEENT: AF 1x1 (No bulging), not pale, no conjunctivitis, anicteric sclera, no injected TM, on ETT No. 4 with 12 cm depth marked
- RS: Tachypnea, mild subcostal retraction, good air entry, clear, equal breath sound both lungs
- CVS: Tachycardia, pulse full, no cyanosis, normal S1S2, no murmur, capillary refill<2sec
- Abdomen: Mild distension, hypoactive bowel sound, soft, no palpable mass, liver span 7 cm, liver 3 cm BRCM, spleen 2 cm BLCM
- MS: No deformities, no edema
- LN: No enlargement
- Genitalia: Normal female type, on Foley catheter
- Neurological examination:
- Consciousness: Stupor (stimulated by pain)
- GCS: E2M4Vt
- Motor: at least gr II all extremities
- Sensory: can’t be evaluated
- Cerebellar: can’t be evaluated
- DTRs: 0 all extremities
- Other: Doll eye’s positive
- Cranial nerve:

CN I: can’t be evaluated

CN II: pupil 3 mmRTLBE

CN III, IV, VI: corneal reflex positive

CN V: corneal reflex positive

CN VII: no facial palsy

CN VIII: can’t be evaluated

CN IX, X: on ETT

CN XI: can’t be evaluated

CN XII: on ETT

**2^nd^ evaluation at day 3**

**Patient’s progression (at day 3)**

- **ผู้ป่วยยังคงซึม มีหายใจเร็ว (ยัง on ventilator)**
- **Heart rate and Blood pressure stable**
- **Urine ออกดี ไม่มีท้องเสีย**
- **Rectal swab culture: Salmonella group C**
- **ได้รับ Meropenem IV, Acyclovir IV (CNS dose)**

**3rd evaluation at discharge date (day 24)**

**Progression at day 3-5**

- On Continuous veno-venous hemodialysis (CVVHD) for 48 hours resulted in improvement of metabolic acidosis

(HCO3 = 33 mmol/l)

**Progression at discharge date (day 10-24)**

| **Date** | **Progression** | **Management** |
| --- | --- | --- |
| Day 10 | ตื่นดี หายใจเอง  Vital signs stable | - on O2 cannula 2 LPM - off NPO - start feeding via NG tube (LF/BM) - off C-line at femoral |
| Day 14 | CSF PCR for Herpes virus : negative | - off O2 cannula - off Acyclovir |
| Day 15 | รับ feed ได้ดี | - step enteral feeding - off Meropenem - off thiamine, off co enzyme Q10 |
| Day 17 | มีไข้ต่ำๆ วันละ 1 ครั้ง  no organ specific signs and symptom  no clinical sepsis | - off C-line at Right internal jugular vein |
| Day 18 | consult nutrition | - Breast feeding - Solid food high calories 3 มื้อ/วัน - add MTV, OTE |
| Day 19 | กินปากได้ ไม่ยอมดูดนม | - consult OT |
| Day 23 | Look well | - Influenza vaccine |
| Day 24 | - Family conference  - Genetic counselling  - Discharge |  |

Laboratory order form – control group

Laboratory order form – intervention group

Case Laboratory results – evaluation 1

| **CBC** | | | |
| --- | --- | --- | --- |
| Test name | Result | Unit | Reference Value |
| **Hct**  **Hb**  **MCV**  **RDW**  **WBC**  **Neutrophil**  **Lymphocyte**  **Monocyte**  **Eosinophil**  **Basophil**  **Platelets** | 32.5  10.4  64.4  18  30,900  67  31  2  0  0  656,000 | %  g/dl  fl  %  /mm3  %  %  %  %  %  /mm^3^ | 44-70  15-24  80-100  11.0-14.5 |

| **Electrolytes** | | | |
| --- | --- | --- | --- |
| Test name | Result | Unit | Reference Value |
| **Na**  **K**  **Cl**  **HCO_3_**  **Anion gap**  **Ca**  **Mg**  **PO_4_** | 138  4  89  2  47  8.79  0.8  4.4 | mmol/L  mmol/L  mmol/L  mmol/L  mmol/L  mg/dL  mmol/L  mg/dL | 133-146   3.4-6.0  96-104   21-28  7-16   8.4-10.2  0.7-1  4-6.5 |

| **Blood sugar** | | | |
| --- | --- | --- | --- |
| Test name | Result | Unit | Reference Value |
| Blood sugar | 61 | mg/dL | >60 |

| **Renal function** | | | |
| --- | --- | --- | --- |
| Test name | Result | Unit | Reference Value |
| **BUN**  **Creatinine** | 28  0.22 | mg/dL  mg/dL | 3-12  0.03-0.5 |

| **Liver function** | | | |
| --- | --- | --- | --- |
| Test name | Result | Unit | Reference Value |
| **Total bilirubin** | 0.62 | mg/dL | 0-13 |
| **Direct bilirubin** | 0.24 | mg/dL | <1 |
| **Total protein** | 5.3 | mg/dL | 4.6-7.4 |
| **Albumin** | 3.48 | g/dL | 2.5-4.9 |
| **AST** | 390 | U/L | 22-71 |
| **ALT** | 117 | U/L | 10-40 |
| **ALP** | 166 | U/L | 48-406 |

| **Coagulogram** |
| --- |
| **PT** 16.5 Sec (13.6)  **PTT** 35.5 Sec (29.1)  **INR** 1.21 |

| **C-reactive protein** |
| --- |
| **Data unavailable** |

| **Hemoculture** |
| --- |
| **Day 1: No bacterial growth** |

| **Serum ammonia** | | | |
| --- | --- | --- | --- |
| Test name | Result | Unit | Reference Value |
| Serum ammonia | 114 | ug/dl | 30-120 |

| **Blood lactate (venous)** | | | |
| --- | --- | --- | --- |
| Test name | Result | Unit | Reference Value |
| Venous lactate | 0.7 | mmol/L | 1.8-2.2 |

| **Urinalysis** | | | |
| --- | --- | --- | --- |
| Test name | Result | Unit | Reference Value |
| Color  Spgr  pH  Protein  Glucose  Ketone  RBC  WBC | Yellow  1.019  5.5  1+  -  1+  20-30  3-5 | /HPF  /HPF | 1.003-1.030  4.6-8.0  -  -  -  0-5  0-5 |

| Comprehensive metabolic testing |
| --- |
| **Amino acids and acylcarnitine profiles**  **Increase C4OH (3-OH butyrylcarnitine) suggests ketosis** |

| **Plasma amino acid** |
| --- |
| Data unavailable |

| **Urine organic acid** |
| --- |
| Increase lactic acid, ketone,  adipic acid excretion in urine |

| **CXR** |
| --- |
| No abnormal infiltration  No cardiac enlargement  Normal bony structure  Normal bowel gas pattern |

| EKG |
| --- |
| Heart rate 120 /min regular  Normal axis  No chamber enlargement by voltage |

| **CT brain**  (Non contrast) |
| --- |
| No structural brain abnormality |


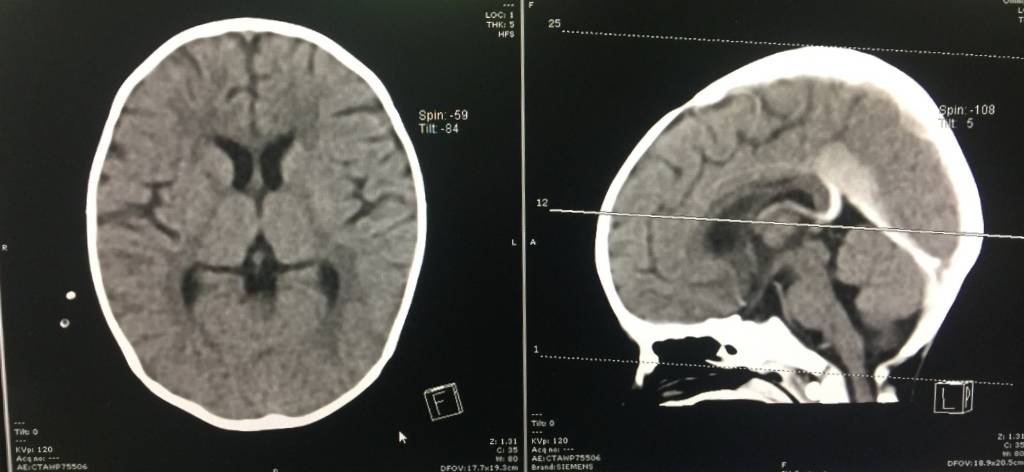


| **MRI brain** |
| --- |
| **Data unavailable** |

| MRS brain |
| --- |
| **Data unavailable** |

| PET scan |
| --- |
| **Data not available** |

| **Continuous EEG monitoring** |
| --- |
| **Rhythmic delta run at left hemisphere**  (no abrupt change in amplitude frequency and distribution, no clinical seizures) |


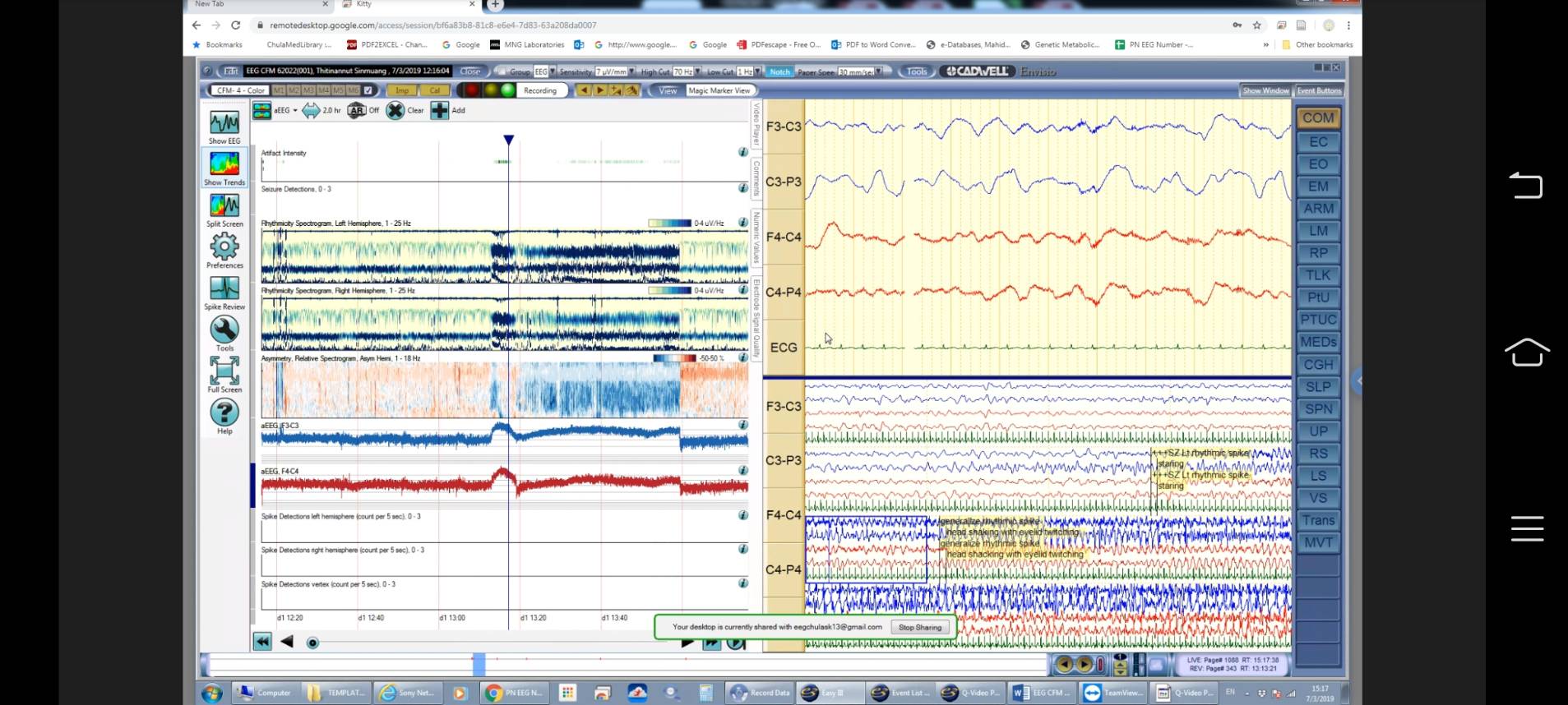


| **Ultrasound brain** |
| --- |
| **No structural brain abnormality** |

| **CSF profile** | | | |
| --- | --- | --- | --- |
| Test name | Result | Unit | Reference Value |
| Color  WBC  RBC  Protein  Sugar | Clear  0  0  normal  normal | /mm3  /mm3  mg/dl  mg/dl | Clear  0-5  0-2 |

| CSF Gram stain |
| --- |
| **No organism** |

| **CSF culture** |
| --- |
| **No bacterial growth** |

| **CSF PCR for Herpes virus** |
| --- |
| **Negative** |

| **CSF amino acid** |
| --- |
| **Data not available** |

| **CSF bacterial antigen test** |
| --- |
| **Negative for**  *N. meningitidis serogroup A, C, Y, W-135*  *Streptococcus pneumoniae*  *Haemophilus influenzae type b*  *Group B Streptococcus species,*  *Escherichia coli* |

| **CSF lactate** |
| --- |
| **1.84 mmol/L (1.8-2.2 mmol/L)** |

| **Skin biopsy** |
| --- |
| **Data unavailable** |

| **Muscle biopsy** |
| --- |
| **Data unavailable** |

| **Chromosome study** |
| --- |
| **Data unavailable** |

| **Chromosome microarray** |
| --- |
| Data not available |

| **Common mitochondrial DNA mutation**  (MELAS/MERF/Leigh syndrome: A3243G, A8344G, T8993G) |
| --- |
| **Data unavailable** |

Laboratory results – evaluation 2

| **CBC** | | | |
| --- | --- | --- | --- |
| Test name | Result | Unit | Reference Value |
| **Hct**  **Hb**  **MCV**  **RDW**  **WBC**  **Neutrophil**  **Lymphocyte**  **Monocyte**  **Eosinophil**  **Basophil**  **Platelets** | 30.2  8.9  70.3  17  18,930  71.5  22.5  5.8  0  0.2  543,000 | %  g/dl  fl  %  /mm3  %  %  %  %  %  /mm^3^ | 44-70  15-24  80-100  11.0-14.5 |

| **Electrolytes** | | | |
| --- | --- | --- | --- |
| Test name | Result | Unit | Reference Value |
| **Na**  **K**  **Cl**  **HCO_3_**  **Anion gap**  **Ca**  **Mg**  **PO_4_** | 145  3.8  89  3  53  6.2  0.55  4.4 | mmol/L  mmol/L  mmol/L  mmol/L  mmol/L  mg/dL  mmol/L  mg/dL | 133-146   3.4-6.0  96-104   21-28  7-16   8.4-10.2  0.7-1  4-6.5 |

| **Blood sugar** | | | |
| --- | --- | --- | --- |
| Test name | Result | Unit | Reference Value |
| Blood sugar | 203 | mg/dL | >60 |

| **Renal function** | | | |
| --- | --- | --- | --- |
| Test name | Result | Unit | Reference Value |
| **BUN**  **Creatinine** | 13  0.2 | mg/dL  mg/dL | 3-12  0.03-0.5 |

| **Liver function** | | | |
| --- | --- | --- | --- |
| Test name | Result | Unit | Reference Value |
| **Total bilirubin** | 0.41 | mg/dL | 0-13 |
| **Direct bilirubin** | 0.37 | mg/dL | <1 |
| **Total protein** | 5.5 | mg/dL | 4.6-7.4 |
| **Albumin** | 3.8 | g/dL | 2.5-4.9 |
| **AST** | 503 | U/L | 22-71 |
| **ALT** | 191 | U/L | 10-40 |
| **ALP** | 194 | U/L | 48-406 |

| **Coagulogram** |
| --- |
| **PT 20.7 Sec (11.0)**  **PTT 54 Sec (25)**  **INR 1.98** |

| **C-reactive protein** |
| --- |
| **Data unavailable** |

| **Hemoculture** |
| --- |
| **Day 3: No bacterial growth** |

| **Serum ammonia** | | | |
| --- | --- | --- | --- |
| Test name | Result | Unit | Reference Value |
| Serum ammonia | 114 | ug/dl | 30-120 |

| **Blood lactate (venous)** | | | |
| --- | --- | --- | --- |
| Test name | Result | Unit | Reference Value |
| Venous lactate | 0.7 | mmol/L | 1.8-2.2 |

| **Urinalysis** | | | |
| --- | --- | --- | --- |
| Test name | Result | Unit | Reference Value |
| Color  Spgr  pH  Protein  Glucose  Ketone  RBC  WBC | Yellow  1.009  6  Trance  2+  1+  50-100  3-5 | /HPF  /HPF | 1.003-1.030  4.6-8.0  -  -  -  0-5  0-5 |

| Comprehensive metabolic testing |
| --- |
| **Amino acids and acylcarnitine profiles**  **Increase C4OH (3-OH butyrylcarnitine) suggests ketosis** |

| **Plasma amino acid** |
| --- |
| Data unavailable |

| **Urine organic acid** |
| --- |
| Increase lactic acid, ketone,  adipic acid excretion in urine |

| **CXR** |
| --- |
| No abnormal infiltration  No cardiac enlargement  Normal bony structure  Normal bowel gas pattern |

| EKG |
| --- |
| Heart rate 120 /min regular  Normal axis  No chamber enlargement by voltage |

| **CT brain**  (Non contrast) |
| --- |
| No structural brain abnormality |


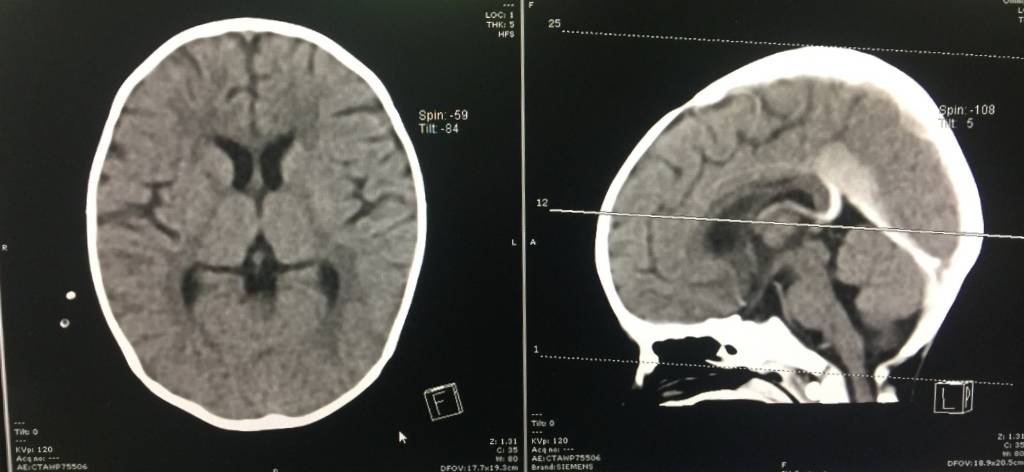


| **MRI brain** |
| --- |
| **Data unavailable** |

| MRS brain |
| --- |
| **Data unavailable** |

| PET scan |
| --- |
| **Data not available** |

| **Continuous EEG monitoring** |
| --- |
| **Data not available** |

| **Ultrasound brain** |
| --- |
| **No structural brain abnormality** |

| **CSF profile** | | | |
| --- | --- | --- | --- |
| Test name | Result | Unit | Reference Value |
| Color  WBC  RBC  Protein  Sugar | Clear  0  0  normal  normal | /mm3  /mm3  mg/dl  mg/dl | Clear  0-5  0-2 |

| CSF Gram stain |
| --- |
| **No organism** |

| **CSF culture** |
| --- |
| **No bacterial growth** |

| **CSF PCR for Herpes virus** |
| --- |
| **Negative** |

| **CSF amino acid** |
| --- |
| **Data not available** |

| **CSF bacterial antigen test** |
| --- |
| **Negative for**  *N. meningitidis serogroup A, C, Y, W-135*  *Streptococcus pneumoniae*  *Haemophilus influenzae type b*  *Group B Streptococcus species,*  *Escherichia coli* |

| **CSF lactate** |
| --- |
| **1.84 mmol/L (1.8-2.2 mmol/L)** |

| **Skin biopsy** |
| --- |
| **Data unavailable** |

| **Muscle biopsy** |
| --- |
| **Data unavailable** |

| **Chromosome study** |
| --- |
| **Data unavailable** |

| **Chromosome microarray** |
| --- |
| Data not available |

| **Common mitochondrial DNA mutation**  (MELAS/MERF/Leigh syndrome: A3243G, A8344G, T8993G) |
| --- |
| **Data unavailable** |

Laboratory results – evaluation 3

| **CBC** | | | |
| --- | --- | --- | --- |
| Test name | Result | Unit | Reference Value |
| **Hct**  **Hb**  **MCV**  **RDW**  **WBC**  **Neutrophil**  **Lymphocyte**  **Monocyte**  **Eosinophil**  **Basophil**  **Platelets** | 35.5  11.5  74.7  29.5  7,070  32  58.8  7.4  0.8  1  352,000 | %  g/dl  fl  %  /mm3  %  %  %  %  %  /mm^3^ | 44-70  15-24  80-100  11.0-14.5 |

| **Electrolytes** | | | |
| --- | --- | --- | --- |
| Test name | Result | Unit | Reference Value |
| **Na**  **K**  **Cl**  **HCO_3_**  **Anion gap**  **Ca**  **Mg**  **PO_4_** | 139  4.7  107  20  12  10.7  0.87  5.8 | mmol/L  mmol/L  mmol/L  mmol/L  mmol/L  mg/dL  mmol/L  mg/dL | 133-146   3.4-6.0  96-104   21-28  7-16   8.4-10.2  0.7-1  4-6.5 |

| **Blood sugar** | | | |
| --- | --- | --- | --- |
| Test name | Result | Unit | Reference Value |
| Blood sugar | 87 | mg/dL | >60 |

| **Renal function** | | | |
| --- | --- | --- | --- |
| Test name | Result | Unit | Reference Value |
| **BUN**  **Creatinine** | 9  0.21 | mg/dL  mg/dL | 3-12  0.03-0.5 |

| **Liver function** | | | |
| --- | --- | --- | --- |
| Test name | Result | Unit | Reference Value |
| **Total bilirubin** | 0.36 | mg/dL | 0-13 |
| **Direct bilirubin** | 0.2 | mg/dL | <1 |
| **Total protein** | 6.9 | mg/dL | 4.6-7.4 |
| **Albumin** | 4.4 | g/dL | 2.5-4.9 |
| **AST** | 68 | U/L | 22-71 |
| **ALT** | 65 | U/L | 10-40 |
| **ALP** | 174 | U/L | 48-406 |

| **Coagulogram** |
| --- |
| **PT 14.7 Sec (11.0)**  **PTT 31.4 Sec (25)**  **INR 1.37** |

| **C-reactive protein** |
| --- |
| **Data unavailable** |

| **Hemoculture** |
| --- |
| **Day 7: No bacterial growth** |

| **Serum ammonia** | | | |
| --- | --- | --- | --- |
| Test name | Result | Unit | Reference Value |
| Serum ammonia | 114 | ug/dl | 30-120 |

| **Blood lactate (venous)** | | | |
| --- | --- | --- | --- |
| Test name | Result | Unit | Reference Value |
| Venous lactate | 0.7 | mmol/L | 1.8-2.2 |

| **Urinalysis** |
| --- |
| Data unavailable |

| Comprehensive metabolic testing |
| --- |
| **Amino acids and acylcarnitine profiles**  **Increase C4OH (3-OH butyrylcarnitine) suggests ketosis** |

| **Plasma amino acid** |
| --- |
| Data unavailable |

| **Urine organic acid** |
| --- |
| Increase lactic acid, ketone,  adipic acid excretion in urine |

| **CXR** |
| --- |
| No abnormal infiltration  No cardiac enlargement  Normal bony structure  Normal bowel gas pattern |

| EKG |
| --- |
| Heart rate 120 /min regular  Normal axis  No chamber enlargement by voltage |

| **CT brain**  (Non contrast) |
| --- |
| No structural brain abnormality |


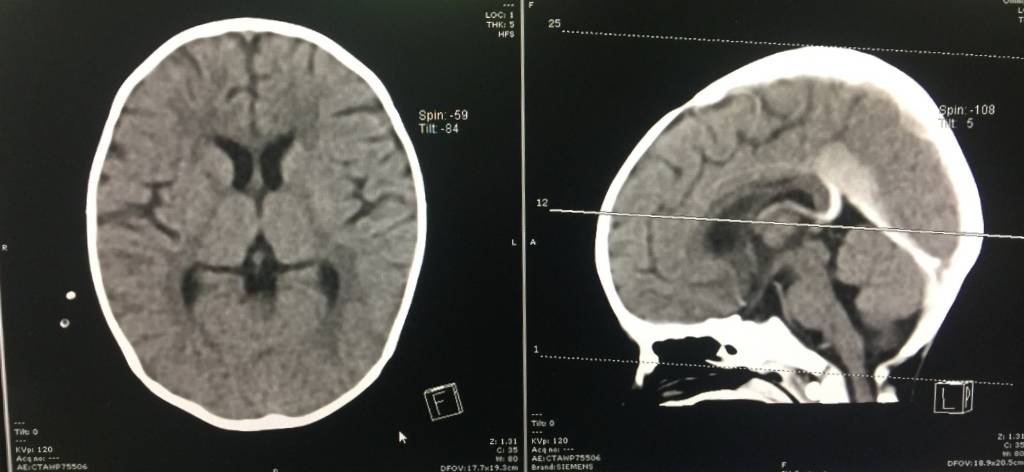


| **MRI brain** |
| --- |
| **Data unavailable** |

| MRS brain |
| --- |
| **Data unavailable** |

| PET scan |
| --- |
| **Data not available** |

| **Continuous EEG monitoring** |
| --- |
| **Data not available** |

| **Ultrasound brain** |
| --- |
| **No structural brain abnormality** |

| **CSF profile** | | | |
| --- | --- | --- | --- |
| Test name | Result | Unit | Reference Value |
| Color  WBC  RBC  Protein  Sugar | Clear  0  0  normal  normal | /mm3  /mm3  mg/dl  mg/dl | Clear  0-5  0-2 |

| CSF Gram stain |
| --- |
| **No organism** |

| **CSF culture** |
| --- |
| **No bacterial growth** |

| **CSF PCR for Herpes virus** |
| --- |
| **Negative** |

| **CSF amino acid** |
| --- |
| **Data not available** |

| **CSF bacterial antigen test** |
| --- |
| **Negative for**  *N. meningitidis serogroup A, C, Y, W-135*  *Streptococcus pneumoniae*  *Haemophilus influenzae type b*  *Group B Streptococcus species,*  *Escherichia coli* |

| **CSF lactate** |
| --- |
| **1.84 mmol/L (1.8-2.2 mmol/L)** |

| **Skin biopsy** |
| --- |
| **Data unavailable** |

| **Muscle biopsy** |
| --- |
| **Data unavailable** |

| **Chromosome study** |
| --- |
| **Data unavailable** |

| **Chromosome microarray** |
| --- |
| Data not available |

| **Common mitochondrial DNA mutation**  (MELAS/MERF/Leigh syndrome: A3243G, A8344G, T8993G) |
| --- |
| **Data unavailable** |

Laboratory results - rWES

| **Rapid exome sequencing** |
| --- |
| Two compound heterozygous variants in the **HMGCS2 gene** were identified in the patient by trio-rWES analysis.   - The novel heterozygous missense **pathogenic variant** (c.1502G>C, p.Arg501Pro)   was inherited from her father.   - The known heterozygous missense **pathogenic variant** (c. 520T>C, p.Phe174Leu) was inherited from her mother. |

| **HMGCS2 gene review** |
| --- |
| - Mutations in this gene are known to cause **mitochondrial 3-hydroxy-3-methylglutaryl CoA synthase deficiency**   **Suggestion**   - Severe metabolic acidosis could be corrected only by **renal replacement therapy**. - Avoid fasting, hypoglycemia - **Emergency letter** to prevent the critical complications of diarrhea, vomiting and poor feeding should be provided - **High carbohydrate, low fat, low protein diets** should be recommended - Genetic counselling: **autosomal recessive** recurrence risk in sibling of proband 25%, recurrence risk in offspring of proband depends on carrier status of spouse |

4.1 Diagnostic sheet – evaluation 1

4.2 Diagnostic sheet – evaluation 2

4.3 Diagnostic sheet – evaluation 3

Doctor order sheet – evaluation 1

Doctor order sheet – evaluation 2

Doctor order sheet – evaluation 3

Case #2

**Case scenario 2**

**ผู้ป่วยรายนี้มีทั้งหมด 3 evaluations**

evaluation ที่ 1 at first day of admission

evaluation ที่ 2 at day 2 of admission

evaluation ที่ 3 at day 3 of admission (discharge date)

- **1st evaluation at first day of admission**
- ผู้ป่วยเด็กหญิงไทย อายุ 5 ปี
- ซึมลง 4 ชั่วโมง ก่อนมาโรงพยาบาล
- 1 วันก่อนมาโรงพยาบาล มีไข้ ไอ มีน้ำมูกใสๆ ไม่ซึม ทานอาหารได้ ปัสสาวะอุจจาระปกติ พูดคุยได้
- 4 ชั่วโมงก่อนมาโรงพยาบาล กระสับกระส่าย ซึมลง ไม่ทำตามสั่ง ยังคงมีไข้ต่ำๆอยู่ คลื่นไส้ ไม่อาเจียน
- ที่ห้องฉุกเฉินของโรงพยาบาล
- ซึมลง เรียกไม่ลืมตา ไม่ทำตามสั่ง ไม่พูด
- Vital signs BT 37.5 C, BP 107/78 mmHg, PR 128/min, RR 28/min, SpO2 98%,
- Neuro: Stupor, E2V2M4, pupil 4 mm RTLBE, equal movement both extremities, stiff neck negative
- Management: Endotracheal intubation, Admit PICU
- ตอนอายุ 3 ปี (2 ปีที่แล้ว) เคย admit สงสัย Acute disseminated encephalomyelitis
- ในตอนนั้นมีประวัติว่าขณะอยู่โรงเรียน มีอาการเกร็ง ตาลอยเป็นระยะ ร่วมกับน้ำลายไหล ไปโรงพยาบาล ระหว่าง admit ซึมลง ไม่รู้สึกตัว กระสับกระส่าย ไม่ทำตามคำสั่ง
- Investigation ในครั้งนั้น
- Normal CSF profies, CSF culture – No bacterial growth
- Serum mycoplasma IgM: positive
- MRI Brain + MRA: Multiple small hyper SI lesion scattered at subcortical and deep white matter of bilateral frontoparietal lobe
- Management: IV cefotaxime 4 days, Acyclovir 3 days, Azithromicin 3 days
- Management ในตอนนั้น cefotaxime IV 4 days, Acyclovir 3 days, Azithromicin 3 days, NPO, IV Fluid
- Progression ตื่นดีภายใน 24 ชั่วโมง
- Follow up หลังจากนั้น 2 เดือน อาการดี ไม่มีความผิดปกติ
- ปฏิเสธโรคประจำตัวอื่นๆ
- ปฏิเสธประวัติแพ้ยา และ/หรือ อาหาร
- ปฏิเสธ drug/toxic substance ingestion
- ปฏิเสธประวัติอุบัติเหตุ
- ประวัติคลอด
- Term G1/2 Normal Labor BW 2,510 g (P25-50th) AGA APGAR 9,10
- มารดาฝากครรภ์ปกติ serology negative all ไม่มีไข้/น้ำเดินก่อนคลอด
- กินแต่นมแม่จนถึงอายุ 6 เดือน
- เริ่มให้นมผงตั้งแต่อายุ 6 เดือน และตอนนี้กินข้าว 3 มื้อ (เลือกกินอาหาร ไม่ค่อยกินเนื้อสัตว์ ชอบกินข้าวกับผัก)
- วัคซีนครบตาม EPI
- พัฒนาการ
- เรียนอนุบาล 2 ครูบอกว่าเรียนช้ากว่าเพื่อน
- เดิน วิ่ง กินข้าวเองได้ ติดกระดุมเสื้อเองได้บ้าง

**Pedigree of three generations**

- ปฏิเสธการแต่งงานในเครือญาติ
- ปฏิเสธคนในครอบครัว เสียชีวิตไม่ทราบสาเหตุ ชัก หรือ พัฒนาการล่าช้า

**Physical examination**

- GA: A Thai girl, stupor, no abnormal odor
- Weight 15 kg (P10-25), Height 83 cm (P10-25), Head circumference at P3
- Vital signs: BT 38 c, PR 120/min, RR 20/min, BP 108/62 mmHg, SpO2 96%
- Skin: no rash, no petechiae, no abnormal hypo/hyperpigmentation
- HEENT: not pale conjunctiva, anicteric sclera, no lymphadenopathy
- RS: on ET-tube, subcostal retraction, coarse crepitation both lungs
- CVS: Tachycardia, pulse full, no cyanosis, normal S1S2, no murmur, capillary refill<2sec
- Abdomen: Mild distension, normoactive bowel sound, soft, no palpable mass,

liver span 10 cm, liver 6 cm BRCM, spleen cannot be palpated

- Musculoskeletal: No deformities, no edema
- Lymph node: No enlargement
- Genitalia: Normal female type, on Foley catheter
- Neurological examination:
- Consciousness: Stupor
- GCS: E2M4VT
- Motor: at least gr II all extremities
- Sensory: can’t be evaluated
- Cerebellar: can’t be evaluated
- DTRs: 0 all extremities
- Other: Doll eye’s positive
- Cranial nerve:

CN I: can’t be evaluated

CN II: pupil 3 mmRTLBE

CN III, IV, VI: corneal reflex positive

CN V: corneal reflex positive

CN VII: no facial palsy

CN VIII: can’t be evaluated

CN IX, X: on ETT

CN XI: can’t be evaluated

CN XII: on ETT

**2^nd^ evaluation at day 2**

**Management**

- On ventilator setting: PC mode FiO2 0.6 PIP 12 PEEP5 RR 20 Ti 0.7 (then adjust the setting with clinical and ABGs)
- Cefotaxime 1.5 gm IV q 8 hr (300 mg/kg/day)
- Azithromycin sry 4 ml po OD (10mg/kg/day)
- Oseltamivir 45 mg po bid

| **วันที่** | **BP(MAP)** | **Clinical/GCS** | **Lab** | **Management** |
| --- | --- | --- | --- | --- |
| Day 1  9.40 น. | 108/65(75)  PR136 | E2M4VT  Pupil 3 mm RTLBE | - | - Fentanyl 1mcg/kg/hr |
| 11.00 น. | 105/65(75)  PR 136 | - | - | - Access C-line  - CT brain emergency |
| 11.45 น. | 105/73(79)  PR 123 | - | - | - 3%NaCl IV 15 ml/hr  - Lumbar puncture  Open pressure 30 cmH2O  Close pressure 16 cmH2O  - Keep BT 36.5-37.5 C, MAP>70 mmHg  - Monitor EtCO2, Keep 35-40 mmHg  - Head elevate 30 degree |
| 12.00 น. | 118/69(79)  PR125 | Generalized tonic clonic seizure | DTX  91 mg% | - Diazepam 4 mg IV stat |
| 12.30 น. |  | Generalized tonic clonic seizure |  | - Diazepam 4 mg IV stat  - Midazolam 3 mg IV stat  - Levetiracetam 20 mg/kg/dose IV loading  Then 30 mg/kg/day |
| Day 2  0.30 น. | 115/56(69)  PR 119 | Sedate  Pupil 3 mm RTL | - | - Dopamine IV drip 14 mcg/kg/min  - Adrenaline 0.1 mcg/kg/min |
| 4.20 น. | 132/73(86) |  | - | -↓Dopamine IV drip 12 mcg/kg/min |
| 6.00 น. | 133/85(96)  PR105 | Sedate  Pupil 4 mm SRTL | - | -↓Dopamine IV drip 10 mcg/kg/min |
| 8.00 น. | 153/92(106)  PR99 |  | - | -↓Dopamine IV drip 8 mcg/kg/min then wean off dopamine |
| 8.45 น. | 164/73(103)  PR 70 | E1M1VT  Sedate  Pupil 5 mm SRTL | - | -↓Adrenaline 0.08 mcg/kg/min |

**3rd evaluation at discharge date (day 3)**

**Management**

- On ventilator; Monitor EtCO2, Keep 35-40 mmHg
- Head elevate 30 degree
- 3% NaCl IV

| **วันที่** | **BP(MAP)** | **Clinical/GCS** | **Lab** | **Management** |
| --- | --- | --- | --- | --- |
| Day 2  12.45น. | 87/63(63)  PR106 |  |  | - Hold IV  - 3%NaCl 60 ml IV drip in 1 hr(4ml/kg/hr)  - Adrenaline 0.08 mcg/kg/min  - Pulse methylprednisolone 30mg/kg/day |
| 13.00 น. | 94/62(69)  PR105 | Sedate  Pupil 5 mm SRTL |  | -↑Adrenaline 0.12 mcg/kg/min  -Coenzyme Q10  -Off cefotaxime  -Meropenam 120 mg/kg/day |
| 13.35-14.20 น. | 88/52(59)  PR121 |  |  | ↑Adrenaline 0.15-0.23 mcg/kg/min |
| 15.00 น. | 93/56(73)  PR130 |  | DTX440mg% | ↑Adrenaline 0.25 mcg/kg/min  20%albumin 1.3mg/kg IV drip in 4 hr |
| 16.30 น. | 110/63(82)  PR141 | Sedate  Pupil 5 mm NRTL |  | Serum ammonia  Serum PT,PTT,INR |
| Day 2  17.20 น. | 113/62(82)  PR 141 | Sedate  Pupil 5 mm NRTL |  | Levophed 0.04 mcg/kg/min  ↑Adrenaline 0.3 mcg/kg/min  Midazolam drip 2 mcg/kg/min |
| 18.00 น. | 74/45(57)  PR130 | Sedate  Pupil 5 mm NRTL | Ammonia level = 3264  PT 16.2 s  PTT 28.1 s  INR 1.49 | ↑Adrenaline 0.7 mcg/kg/min  ↑Levophed 0.2 mcg/kg/min |
| 18.10 น. | 128/86(104)  PR150 |  |  | ↑Levophed 0.5 mcg/kg/min  ↓Adrenaline 0.2 mcg/kg/min  Consult nephrologist |
| 18.30 น. | 126/78(100)  PR150 |  |  | L-carnitine (100) 1 cab OD  ↓Levophed 0.4mcg/kg/min  Carglumic acid(200) 5 tabs po stat |
| 21.00-21.30 น. | 106/58(78)  PR 127 | Sedate  Pupil 5 mm NRTL |  | ↑Levophed 0.5-0.6 mcg/kg/min  Start CVVHD |
| 23.00 น. | 115/72(92)  PR 129 |  |  |  |
| Day 3  5.00น. | 134/112(122)  PR151 | E1M1VT  Pupil 6 mm NRTLBE |  | ↓Levophed 0.5mcg/kg/min |
| 7.10น. | 132/111 (121) | E1M1VT  Pupil 8 mm NRTLBE | BUN 7 Cr 0.39  Ammonia level = 2765 | ↓Levophed 0.4mcg/kg/min  Off Midazolam drip |
| 11.45น. | 114/97 (106) |  |  | Sodium benzoate 7500 mg po stat  then 1500 mg po q 8 hr |

Laboratory order form – control group

Laboratory order form – intervention group

Laboratory results - evaluation 1

| **CBC** | | | |
| --- | --- | --- | --- |
| Test name | Result | Unit | Reference Value |
| **Hct**  **Hb**  **MCV**  **RDW**  **WBC**  **Neutrophil**  **Lymphocyte**  **Monocyte**  **Eosinophil**  **Basophil**  **Platelets** | 33.4  11.1  69.4  15.5  7,360  68.1  24.4  7.4  0  0.1  480,000 | %  g/dl  fl  %  /mm3  %  %  %  %  %  /mm^3^ | 44-70  15-24  80-100  11.0-14.5 |

| **Electrolytes** | | | |
| --- | --- | --- | --- |
| Test name | Result | Unit | Reference Value |
| **Na**  **K**  **Cl**  **HCO_3_**  **Anion gap**  **Ca**  **Mg**  **PO_4_** | 136  4  104  15  17  8.9  1.04  5.3 | mmol/L  mmol/L  mmol/L  mmol/L  mmol/L  mg/dL  mmol/L  mg/dL | 133-146   3.4-6.0  96-104   21-28  7-16   8.4-10.2  0.7-1  4-6.5 |

| **Blood sugar** | | | |
| --- | --- | --- | --- |
| Test name | Result | Unit | Reference Value |
| Blood sugar | 91 | mg/dL | >60 |

| **Renal function** | | | |
| --- | --- | --- | --- |
| Test name | Result | Unit | Reference Value |
| **BUN**  **Creatinine** | 10  0.43 | mg/dL  mg/dL | 3-12  0.03-0.5 |

| **Liver function** | | | |
| --- | --- | --- | --- |
| Test name | Result | Unit | Reference Value |
| **Total bilirubin** | 0.59 | mg/dL | 0-13 |
| **Direct bilirubin** | 0.34 | mg/dL | <1 |
| **Total protein** | 7.5 | mg/dL | 4.6-7.4 |
| **Albumin** | 3.8 | g/dL | 2.5-4.9 |
| **AST** | 273 | U/L | 22-71 |
| **ALT** | 249 | U/L | 10-40 |
| **ALP** | 183 | U/L | 48-406 |

| **Coagulogram** |
| --- |
| **Data not available** |

| **C-reactive protein** |
| --- |
| **Data unavailable** |

| **Hemoculture** |
| --- |
| **Day 1: No bacterial growth** |

| **Serum ammonia** | | | |
| --- | --- | --- | --- |
| Test name | Result | Unit | Reference Value |
| Serum ammonia | Data not available | ug/dl | 30-120 |

| **Blood lactate (venous)** | | | |
| --- | --- | --- | --- |
| Test name | Result | Unit | Reference Value |
| Venous lactate | 3.3 | mmol/L | 1.8-2.2 |

| **Urinalysis** | | | |
| --- | --- | --- | --- |
| Test name | Result | Unit | Reference Value |
| Data not available | | | |

| Comprehensive metabolic testing |
| --- |
| **Data not available** |

| **Plasma amino acid** |
| --- |
| Data not available |

| **Urine organic acid** |
| --- |
| Data not available |

| **CXR** |
| --- |
| Bilateral perihilar infiltration  No cardiac enlargement  Normal bony structure  Normal bowel gas pattern |


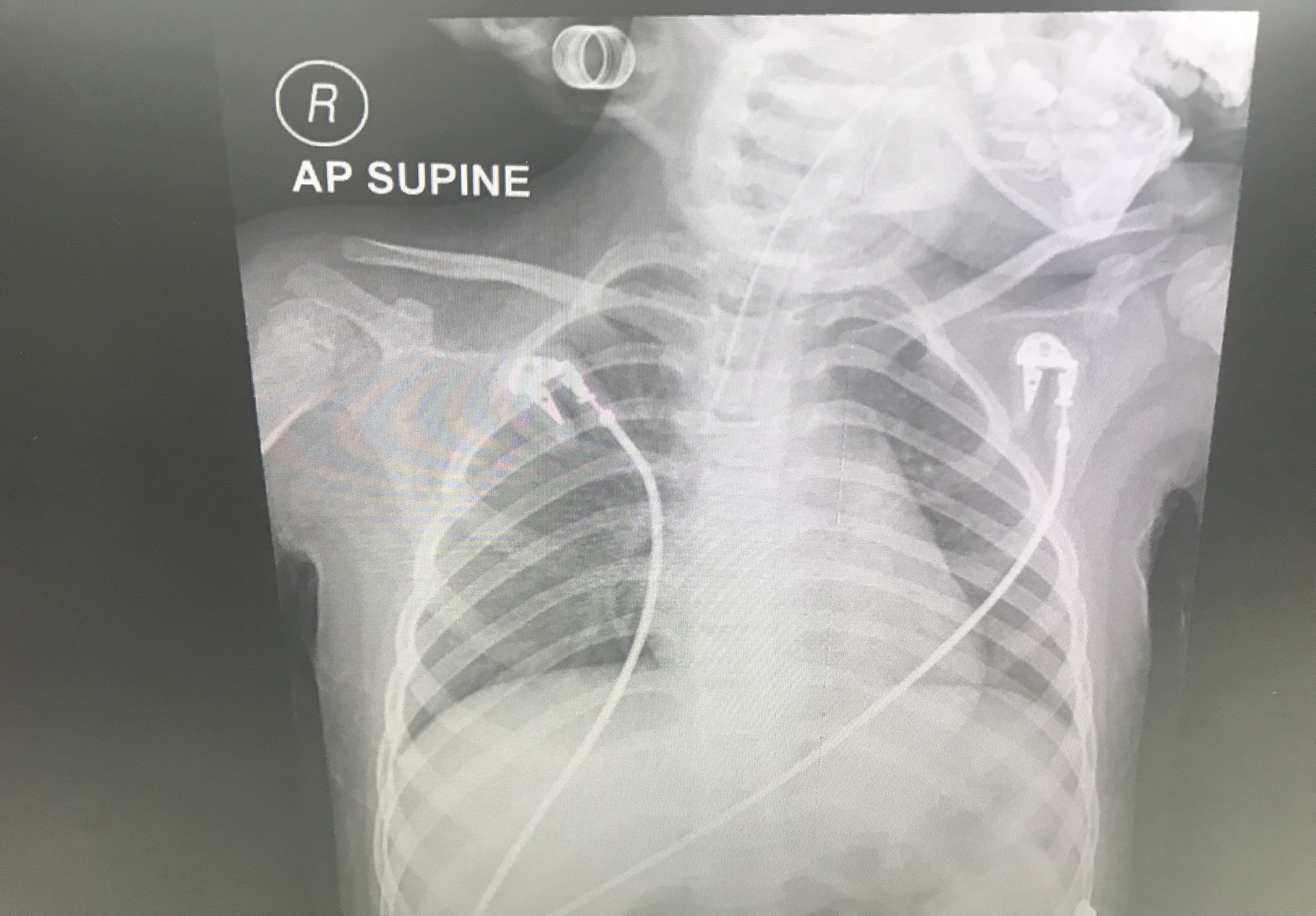


| EKG |
| --- |
| Heart rate 100 /min regular  Normal axis  No chamber enlargement by voltage |

| **CT brain**  (with contrast) |
| --- |
| Mild diffuse brain swelling with leptomeningeal enchancement at bilateral high fronto-parietal lobes |

| **MRI brain** |
| --- |
| **Data unavailable** |

| MRS brain |
| --- |
| **Data unavailable** |

| PET scan |
| --- |
| **Data not available** |

| **Continuous EEG monitoring** |
| --- |
| **Data not available** |

| **Ultrasound brain** |
| --- |
| **Data not available** |

| **CSF profile** | | | |
| --- | --- | --- | --- |
| Test name | Result | Unit | Reference Value |
| Color  WBC  RBC  Protein  Sugar | Light red  28  94,000 (Traumatic tap)  200  72 | /mm3  /mm3  mg/dl  mg/dl | Clear  0-5  0-2 |

| CSF Gram stain |
| --- |
| **No organism** |

| **CSF culture** |
| --- |
| **No bacterial growth** |

| **CSF PCR for Herpes virus** |
| --- |
| **Negative** |

| **CSF amino acid** |
| --- |
| **Data not available** |

| **CSF bacterial antigen test** |
| --- |
| **Negative for**  *N. meningitidis serogroup A, C, Y, W-135*  *Streptococcus pneumoniae*  *Haemophilus influenzae type b*  *Group B Streptococcus species,*  *Escherichia coli* |

| **CSF lactate** |
| --- |
| **Data not available** |

| **Nasal Swab for Rapid**  **Influenza A/B/RSV** |
| --- |
| Influenza A Ag: Positive  Influenza B Ag: Negative  RSV Ag: Negative |

| **Endotracheal aspiration**  **Gram stain and Culture** |
| --- |
| Gram positive cocci in pairs: Many  No bacterial growth in Day 1 |

| **Chromosome study** |
| --- |
| **Data unavailable** |

| **Chromosome microarray** |
| --- |
| Data not available |

| **Common mitochondrial DNA mutation**  (MELAS/MERF/Leigh syndrome: A3243G, A8344G, T8993G) |
| --- |
| **Data unavailable** |

Laboratory results – evaluation 2

| **CBC** | | | |
| --- | --- | --- | --- |
| Test name | Result | Unit | Reference Value |
| **Hct**  **Hb**  **MCV**  **RDW**  **WBC**  **Neutrophil**  **Lymphocyte**  **Monocyte**  **Eosinophil**  **Basophil**  **Platelets** | 31.8  10.7  68.5  15.6  8,540  84.8  11.3  3.8  0  0.1  475,000 | %  g/dl  fl  %  /mm3  %  %  %  %  %  /mm^3^ | 44-70  15-24  80-100  11.0-14.5 |

| **Electrolytes** | | | |
| --- | --- | --- | --- |
| Test name | Result | Unit | Reference Value |
| **Na**  **K**  **Cl**  **HCO_3_**  **Anion gap**  **Ca**  **Mg**  **PO_4_** | 147  3.1  110  20  17  8.9  0.78  2.8 | mmol/L  mmol/L  mmol/L  mmol/L  mmol/L  mg/dL  mmol/L  mg/dL | 133-146   3.4-6.0  96-104   21-28  7-16   8.4-10.2  0.7-1  4-6.5 |

| **Blood sugar** | | | |
| --- | --- | --- | --- |
| Test name | Result | Unit | Reference Value |
| Blood sugar | 472 | mg/dL | >60 |

| **Renal function** | | | |
| --- | --- | --- | --- |
| Test name | Result | Unit | Reference Value |
| **BUN**  **Creatinine** | 4  0.3 | mg/dL  mg/dL | 3-12  0.03-0.5 |

| **Liver function** | | | |
| --- | --- | --- | --- |
| Test name | Result | Unit | Reference Value |
| **Total bilirubin** | 0.59 | mg/dL | 0-13 |
| **Direct bilirubin** | 0.34 | mg/dL | <1 |
| **Total protein** | 7.5 | mg/dL | 4.6-7.4 |
| **Albumin** | 3.8 | g/dL | 2.5-4.9 |
| **AST** | 273 | U/L | 22-71 |
| **ALT** | 249 | U/L | 10-40 |
| **ALP** | 183 | U/L | 48-406 |

| **Coagulogram** |
| --- |
| **PT 16.2**  **Normal 11.3**  **PTT 28.1**  **Normal 17**  **INR 1.49** |

| **C-reactive protein** |
| --- |
| **Data unavailable** |

| **Hemoculture** |
| --- |
| **Day 2: No bacterial growth** |

| **Serum ammonia** | | | |
| --- | --- | --- | --- |
| Test name | Result | Unit | Reference Value |
| Serum ammonia | 3,264 | ug/dl | 30-120 |

| **Blood lactate (venous)** | | | |
| --- | --- | --- | --- |
| Test name | Result | Unit | Reference Value |
| Venous lactate | 1.4 | mmol/L | 1.8-2.2 |

| **Urinalysis** | | | |
| --- | --- | --- | --- |
| Test name | Result | Unit | Reference Value |
| Color  Spgr  pH  Protein  Glucose  Ketone  RBC  WBC | Yellow  1.008  8  1+  1+  3+  0-1  0-1 | /HPF  /HPF | 1.003-1.030  4.6-8.0  -  -  -  0-5  0-5 |

| Comprehensive metabolic testing |
| --- |
| **Increase C4OH (3-OH butyrylcarnitine) suggests ketosis** |

| **Plasma amino acid** |
| --- |
| Non-specific decrease of glycine, alanine, proline, phenylalanine in plasma |

| **Urine organic acid** |
| --- |
| Increase lactic acid, ketone and orotic acid excretion in urine |

| **CXR** |
| --- |
| Bilateral perihilar infiltration  No cardiac enlargement  Normal bony structure  Normal bowel gas pattern |


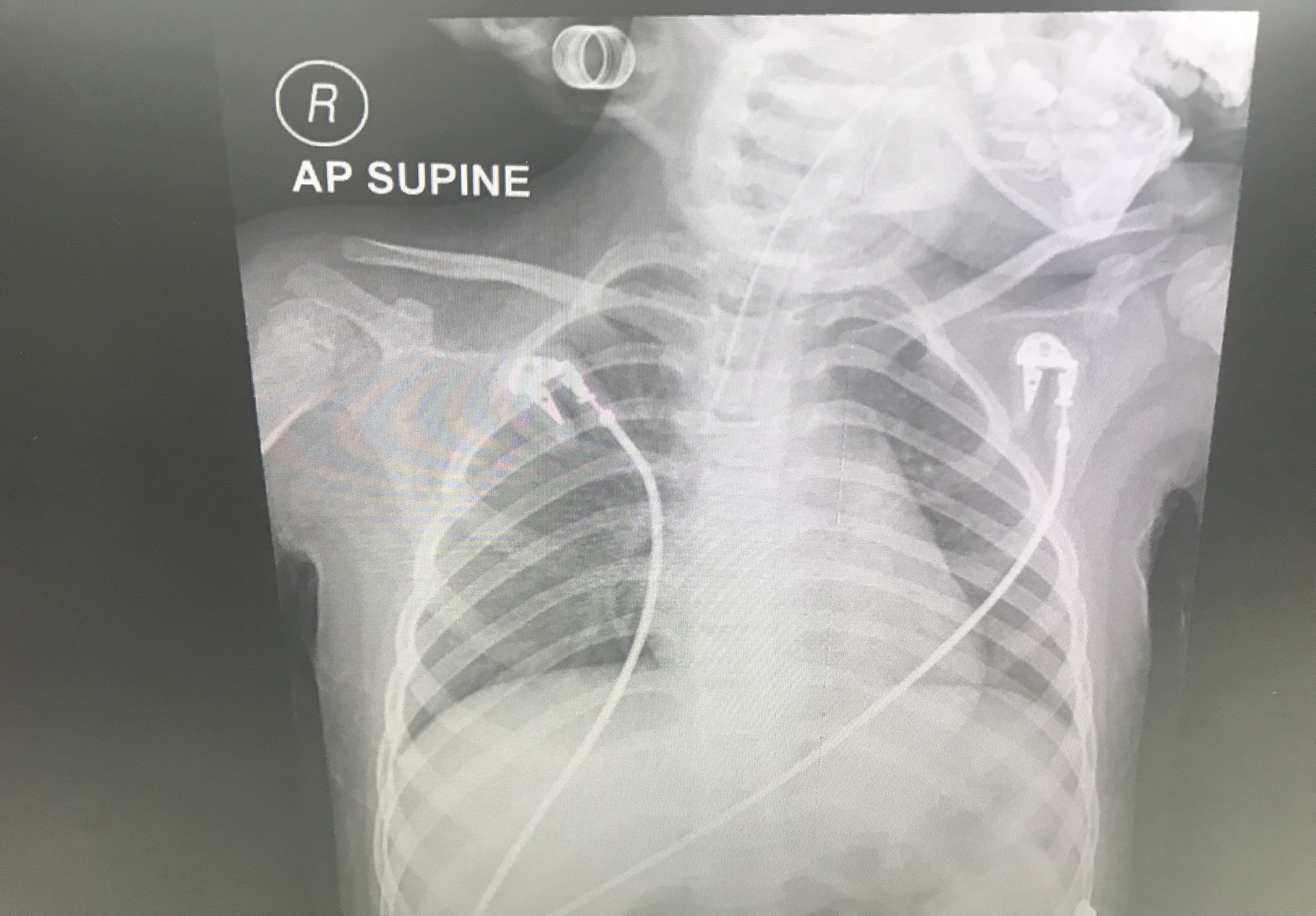


| EKG |
| --- |
| Heart rate 70 /min regular  Normal axis  No chamber enlargement by voltage |

| **CT brain**  (with contrast) |
| --- |
| - Bilateral tonsillar herniation and central herniation - Severe HIE/anoxic brain injury |

| **MRI brain** |
| --- |
| **Data unavailable** |

| MRS brain |
| --- |
| **Data unavailable** |

| PET scan |
| --- |
| **Data not available** |

| **Continuous EEG monitoring** |
| --- |
| **Data not available** |

| **Ultrasound brain** |
| --- |
| **Data not available** |

| **CSF profile** | | | |
| --- | --- | --- | --- |
| Test name | Result | Unit | Reference Value |
| Color  WBC  RBC  Protein  Sugar | Light red  28  94,000 (Traumatic tap)  200  72 | /mm3  /mm3  mg/dl  mg/dl | Clear  0-5  0-2 |

| CSF Gram stain |
| --- |
| **No organism** |

| **CSF culture** |
| --- |
| **No bacterial growth** |

| **CSF PCR for Herpes virus** |
| --- |
| **Negative** |

| **CSF amino acid** |
| --- |
| **Data not available** |

| **CSF bacterial antigen test** |
| --- |
| **Negative for**  *N. meningitidis serogroup A, C, Y, W-135*  *Streptococcus pneumoniae*  *Haemophilus influenzae type b*  *Group B Streptococcus species,*  *Escherichia coli* |

| **CSF lactate** |
| --- |
| **Data not available** |

| **Nasal Swab for Rapid**  **Influenza A/B/RSV** |
| --- |
| Influenza A Ag: Positive  Influenza B Ag: Negative  RSV Ag: Negative |

| **Endotracheal aspiration**  **Gram stain and Culture** |
| --- |
| Gram positive cocci in pairs: Many  No bacterial growth |

| **Chromosome study** |
| --- |
| **Data unavailable** |

| **Chromosome microarray** |
| --- |
| Data not available |

| **Common mitochondrial DNA mutation**  (MELAS/MERF/Leigh syndrome: A3243G, A8344G, T8993G) |
| --- |
| **Data unavailable** |

Laboratory results – evaluation 3

Laboratory results – rWES

Diagnostic sheet – evaluation 1

Diagnostic sheet – evaluation 2

Diagnostic sheet – evaluation 3

Doctor order sheet – evaluation 1

Doctor order sheet – evaluation 2

Doctor order sheet – evaluation 3

Case #3

**Case scenario 3**

**ผู้ป่วยรายนี้มีทั้งหมด 3 evaluations**

evaluation ที่ 1 at first day of admission

evaluation ที่ 2 at day 17 of admission

evaluation ที่ 3 at day 37 of admission (discharge date)

- **1st evaluation at first day of admission**
- **History**
- A 1-year-old Thai girl
- Chief complaint: fever and diarrhea for 2 days

- **Past history**
- At 7 months old, she was admitted to local hospital due to septic shock
- Perinatal history
  - Term GA 37 weeks, normal labor, AGA, no complication after birth
  - Maternal serology: anti-HIV negative, HBsAg negative, VDRL nonreactive
  - Umbilical cord separation at the age of 2 weeks old
- Immunization
  - BCG HBV 1, Rotarix 2, DTaP-HB-IPV-Hib3, MMR1, JE1, Influenza2

**Family history**

- No history of consanguinity
- No family history of unknown death, inborn errors of metabolism

**Physical examination**

- V/S: BT 40 ºC, BP 106/55 mmHg, HR 200 bpm, RR 70/min, SpO2 100%
- Measurement: body weight 9 kg (P25-50), height 73 cm (P10-25)
- GA: A Thai girl, irritable, no dysmorphic feature
- Skin: normal BCG scar at left arm
- Lymph node: no palpable lymphadenopathy
- HEENT: pale conjunctivae, anicteric sclerae, tonsils 1+ both sides, no exudate,
  no oral thrush, dry lips, sunken eyeballs
- CVS: normal S1S2, no murmur, bounding pulse, capillary refill 2 seconds, tachycardia, regular rhythm
- Lung: tachypnea, subcostal and intercostal retraction, good air entry, fine crepitation both lungs, no wheezing
- Abdomen: no distention, normoactive bowel sound, soft, no tenderness, liver 3 FB below RCM, liver span 8 cm, spleen cannot be palpated
- Neuro: Kernig and Brudzinski’s negative

**2^nd^ evaluation at day 17**

**Management**

- NSS 20 ml/kg IV total 3 doses
- Adrenaline 0.05 mcg/kg/min IV
- Ceftriaxone 72 mg/kg/day IV

Then switch to Meropenem IV, Amikacin IV, Oseltamivir Oral,

Azithromycin Oral, TMP-SMX Oral

- Intubation
- Intravenous Immunoglobulin (IVIG)
- Correct serum potassium level

**3rd evaluation at discharge date (day 37)**

**Management**

- Stop TMP-SMX
- **Drug provocation test:** TMP-SMX oral 2.5 mg/kg/dose of TMP
- 9 hours after administration: generalized MP rash at trunk and extremities
- 2 days after administration: resolution of rash
- **Antibacterial prophylaxis:** ciprofloxacin

Laboratory order form – control group

Laboratory order form – intervention group

Laboratory results – evaluation 1

| **CBC** | | | |
| --- | --- | --- | --- |
| Test name | Result | Unit | Reference Value |
| **Hct**  **Hb**  **MCV**  **RDW**  **WBC**  **Neutrophil**  **Lymphocyte**  **Monocyte**  **Eosinophil**  **Basophil**  **Platelets** | 18  6.1  52.1  21.5  6,030  46  46  8  0  0  182,000 | %  g/dl  fl  %  /mm3  %  %  %  %  %  /mm^3^ | 44-70  15-24  80-100  11.0-14.5 |

| **Electrolytes** | | | |
| --- | --- | --- | --- |
| Test name | Result | Unit | Reference Value |
| **Na**  **K**  **Cl**  **HCO_3_**  **Anion gap**  **Ca**  **Mg**  **PO_4_** | 137  2.6  112  10  15  9.0  1.0  4.3 | mmol/L  mmol/L  mmol/L  mmol/L  mmol/L  mg/dL  mmol/L  mg/dL | 133-146   3.4-6.0  96-104   21-28  7-16   8.4-10.2  0.7-1  4-6.5 |

| **Blood sugar** | | | |
| --- | --- | --- | --- |
| Test name | Result | Unit | Reference Value |
| Blood sugar | 88 | mg/dL | >60 |

| **Renal function** | | | |
| --- | --- | --- | --- |
| Test name | Result | Unit | Reference Value |
| **BUN**  **Creatinine** | 9  0.3 | mg/dL  mg/dL | 3-12  0.03-0.5 |

| **Liver function** | | | |
| --- | --- | --- | --- |
| Test name | Result | Unit | Reference Value |
| **Total bilirubin** | 1.4 | mg/dL | 0-13 |
| **Direct bilirubin** | 1.0 | mg/dL | <1 |
|  |  |  |  |
| **Albumin**  **Globulin** | 2.1  1.7 | g/dL  g/dl | 2.5-4.9  2-3.3 |
| **AST** | 123 | U/L | 22-71 |
| **ALT** | 40 | U/L | 10-40 |
| **ALP** | 69 | U/L | 48-406 |

| **Coagulogram** |
| --- |
| **PT 23.6 (11.5-15.3)**  **PTT 50.7 (35.1-46.3)**  **INR 2.17** |

| **D-dimer (mg/L)** |
| --- |
| **>35.2 (0.11-0.42)** |

| **Hemoculture** |
| --- |
| **Day 1: Chromobacterium violaceum** |

| **Serum ammonia** | | | |
| --- | --- | --- | --- |
| Test name | Result | Unit | Reference Value |
| Serum ammonia | Data not available | ug/dl | 30-120 |

| **Blood lactate (venous)** | | | |
| --- | --- | --- | --- |
| Test name | Result | Unit | Reference Value |
| Venous lactate | 3 | mmol/L | 1.8-2.2 |

| **Urinalysis** | | | |
| --- | --- | --- | --- |
| Test name | Result | Unit | Reference Value |
| Color  Spgr  pH  Protein  Glucose  Ketone  RBC  WBC | Yellow  1.025  6.5  neg  neg  neg  0-1  0-1 | /HPF  /HPF | 1.003-1.030  4.6-8.0  -  -  -  0-5  0-5 |

| **Flow cytometry analysis** | | |
| --- | --- | --- |
|  | **Age 1 year 2 months** | **Normal range** |
| Absolute total lymphocyte (cell/mcl) | 12370 ↑ | 2180-8270 |
| %Total lymphocyte | 69.3 ⟷ | 44-72 |
| Absolute CD3(cell/mcl) | 6680 ↑ | 1460-5440 |
| %CD3 | 54.0 ⟷ | 53-81 |
| Absolute CD4(cell/mcl) | 3340 ⟷ | 1020-3600 |
| %CD4 | 27.0 ↓ | 31-54 |
| Absolute CD8(cell/mcl) | 3093 ↑ | 570-2230 |
| %CD8 | 25 ⟷ | 16-38 |
| Absolute CD19(cell/mcl) | 4206 ↑ | 500-1500 |
| %CD19 | 34 ↑ | 19-31 |
| Absolute CD16+56(cell/mcl) | 1484 ↑ | 59-1178 |
| %CD56 | 12 ⟷ | 3-30 |

| **Immunoglobulin level** | | |
| --- | --- | --- |
|  | **Age 1 year (before IVIG)** | **Normal range** |
| **IgG (mg/dl)** | 1090 ⟷ | 223-1099 |
| **IgM(mg/dl)** | 104 ↑ | 8-100 |
| **IgA(mg/dl)** | 174 ↑ | 1-73 |

| **Dihydrorhodamine test (DHR)** |
| --- |
| Positive |


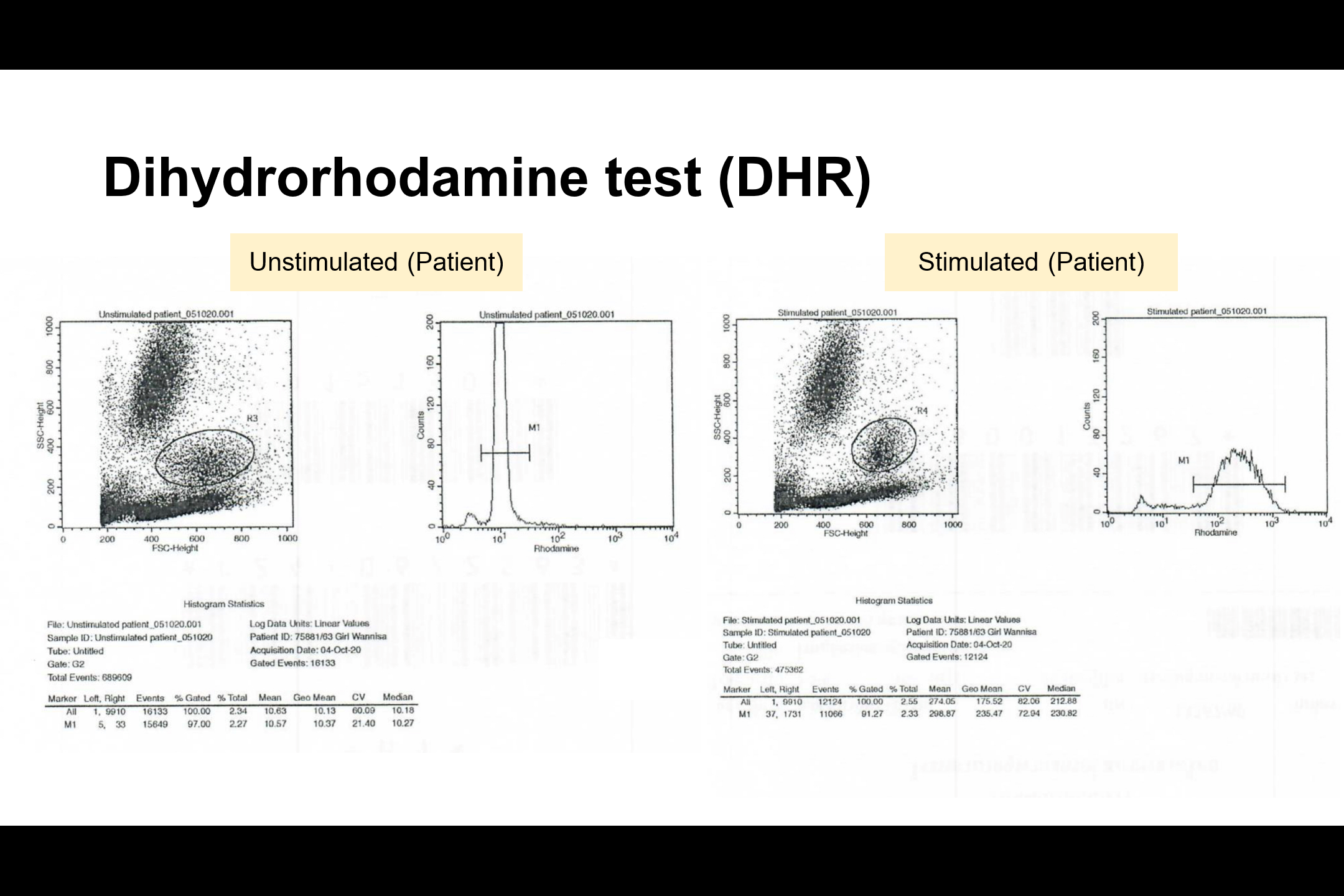


| **CXR** |
| --- |
| Patchy infiltration seen as below image  Hepatomegaly  No cardiac enlargement  Normal bony structure  Normal bowel gas pattern  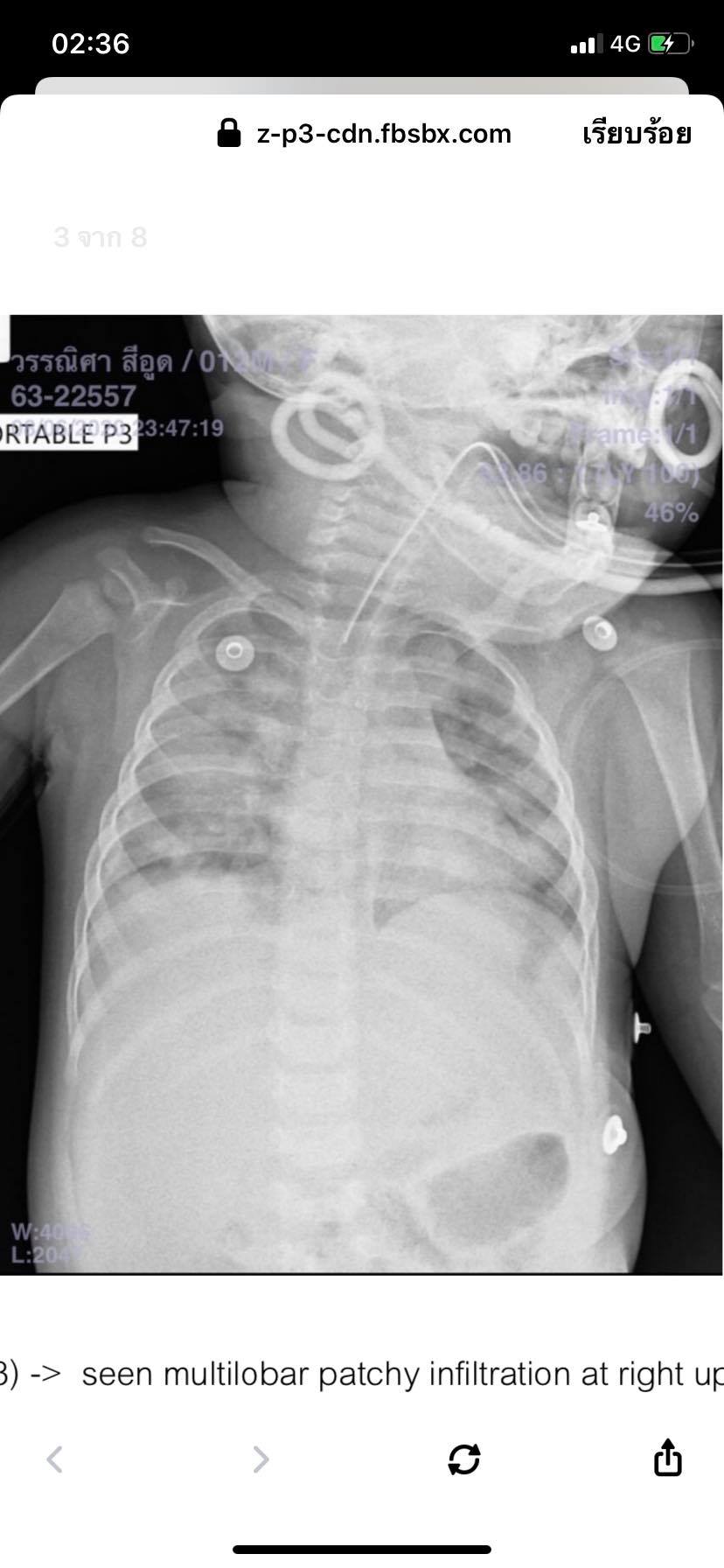 |

| EKG |
| --- |
| Heart rate 90 /min regular  Normal axis  No chamber enlargement by voltage |

| **CT brain**  (with contrast) |
| --- |
| Data unavailable |

| **MRI brain** |
| --- |
| **Data unavailable** |

| MRS brain |
| --- |
| **Data unavailable** |

| PET scan |
| --- |
| **Data not available** |

| Ultrasound whole abdomen |
| --- |
| - Hepatomegaly, normal parenchymal echogenicity - Multiple tiny liver cysts scattering in both hepatic lobes are observed 2-4 mm. in size - Multiple ill-defined hypoechoic lesions in partially visualized spleen are seen 3 mm. to 1 cm. in size - Impression: multiple microabcesses at liver and spleen |

| **Ultrasound brain** |
| --- |
| **Data not available** |

| **CSF profile** | | | |
| --- | --- | --- | --- |
| Test name | Result | Unit | Reference Value |
| Color  WBC  RBC  Protein  Sugar | Clear  0  0  normal  normal | /mm3  /mm3  mg/dl  mg/dl | Clear  0-5  0-2 |

| CSF Gram stain |
| --- |
| **No organism** |

| **CSF culture** |
| --- |
| **No bacterial growth** |

| **CSF PCR for Herpes virus** |
| --- |
| **Negative** |

| **CSF amino acid** |
| --- |
| **Data not available** |

| **CSF bacterial antigen test** |
| --- |
| **Negative for**  *N. meningitidis serogroup A, C, Y, W-135*  *Streptococcus pneumoniae*  *Haemophilus influenzae type b*  *Group B Streptococcus species,*  *Escherichia coli* |

| **CSF lactate** |
| --- |
| **Data not available** |

| **Nasal Swab for Rapid**  **Influenza A/B/RSV** |
| --- |
| Influenza A Ag: Negative  Influenza B Ag: Negative  RSV Ag: Negative |

| **Endotracheal aspiration**  **Gram stain and Culture** |
| --- |
| Tracheal suction culture: S.pneumoniae |

| **Stool exam and culture** |
| --- |
| RBC 0-1 cells/HPF  WBC 0-1 cells/HPF  Pasasite not seen  No bacterial growth |

| **Chromosome microarray** |
| --- |
| Data not available |

| **Common mitochondrial DNA mutation**  (MELAS/MERF/Leigh syndrome: A3243G, A8344G, T8993G) |
| --- |
| **Data unavailable** |

Laboratory results – evaluation 2

| **CBC** | | | |
| --- | --- | --- | --- |
| Test name | Result | Unit | Reference Value |
| **Hct**  **Hb**  **MCV**  **RDW**  **WBC**  **Neutrophil**  **Lymphocyte**  **Monocyte**  **Eosinophil**  **Basophil**  **Platelets** | 25  8.6  62.3  26.3  6,540  27  55  11  7 (457)  0  280,000 | %  g/dl  fl  %  /mm3  %  %  %  %  %  /mm^3^ | 44-70  15-24  80-100  11.0-14.5 |

| **Electrolytes** | | | |
| --- | --- | --- | --- |
| Test name | Result | Unit | Reference Value |
| **Na**  **K**  **Cl**  **HCO_3_**  **Anion gap**  **Ca**  **Mg**  **PO_4_** | 136  5.2  104  20  12  8.9  0.8  4.8 | mmol/L  mmol/L  mmol/L  mmol/L  mmol/L  mg/dL  mmol/L  mg/dL | 133-146   3.4-6.0  96-104   21-28  7-16   8.4-10.2  0.7-1  4-6.5 |

| **Blood sugar** | | | |
| --- | --- | --- | --- |
| Test name | Result | Unit | Reference Value |
| Blood sugar | 79 | mg/dL | >60 |

| **Renal function** | | | |
| --- | --- | --- | --- |
| Test name | Result | Unit | Reference Value |
| **BUN**  **Creatinine** | 4  0.27 | mg/dL  mg/dL | 3-12  0.03-0.5 |

| **Liver function** | | | |
| --- | --- | --- | --- |
| Test name | Result | Unit | Reference Value |
| **Total bilirubin** | 0.3 | mg/dL | 0-13 |
| **Direct bilirubin** | 0.1 | mg/dL | <1 |
|  |  |  |  |
| **Albumin**  **Globulin** | 4.1  2.6 | g/dL  g/dl | 2.5-4.9  2-3.3 |
| **AST** | 152 | U/L | 22-71 |
| **ALT** | 93 | U/L | 10-40 |
| **ALP** | 250 | U/L | 48-406 |

| **Coagulogram** |
| --- |
| Data not available |

| **D-dimer (mg/L)** |
| --- |
| Data not available |

| **Hemoculture** |
| --- |
| Data not available |

| **Serum ammonia** | | | |
| --- | --- | --- | --- |
| Test name | Result | Unit | Reference Value |
| Serum ammonia | Data not available | ug/dl | 30-120 |

| **Blood lactate (venous)** | | | |
| --- | --- | --- | --- |
| Test name | Result | Unit | Reference Value |
| Venous lactate | 3 | mmol/L | 1.8-2.2 |

| **Urinalysis** | | | |
| --- | --- | --- | --- |
| Test name | Result | Unit | Reference Value |
| Color  Spgr  pH  Protein  Glucose  Ketone  RBC  WBC | Yellow  1.025  6.5  neg  neg  neg  0-1  0-1 | /HPF  /HPF | 1.003-1.030  4.6-8.0  -  -  -  0-5  0-5 |

| **Flow cytometry analysis** | | |
| --- | --- | --- |
|  | **Age 1 year 2 months** | **Normal range** |
| Absolute total lymphocyte (cell/mcl) | 12370 ↑ | 2180-8270 |
| %Total lymphocyte | 69.3 ⟷ | 44-72 |
| Absolute CD3(cell/mcl) | 6680 ↑ | 1460-5440 |
| %CD3 | 54.0 ⟷ | 53-81 |
| Absolute CD4(cell/mcl) | 3340 ⟷ | 1020-3600 |
| %CD4 | 27.0 ↓ | 31-54 |
| Absolute CD8(cell/mcl) | 3093 ↑ | 570-2230 |
| %CD8 | 25 ⟷ | 16-38 |
| Absolute CD19(cell/mcl) | 4206 ↑ | 500-1500 |
| %CD19 | 34 ↑ | 19-31 |
| Absolute CD16+56(cell/mcl) | 1484 ↑ | 59-1178 |
| %CD56 | 12 ⟷ | 3-30 |

| **Immunoglobulin level** | | |
| --- | --- | --- |
|  | **Age 1 year (before IVIG)** | **Normal range** |
| **IgG (mg/dl)** | 1090 ⟷ | 223-1099 |
| **IgM(mg/dl)** | 104 ↑ | 8-100 |
| **IgA(mg/dl)** | 174 ↑ | 1-73 |

| **Dihydrorhodamine test (DHR)** |
| --- |
| Positive |


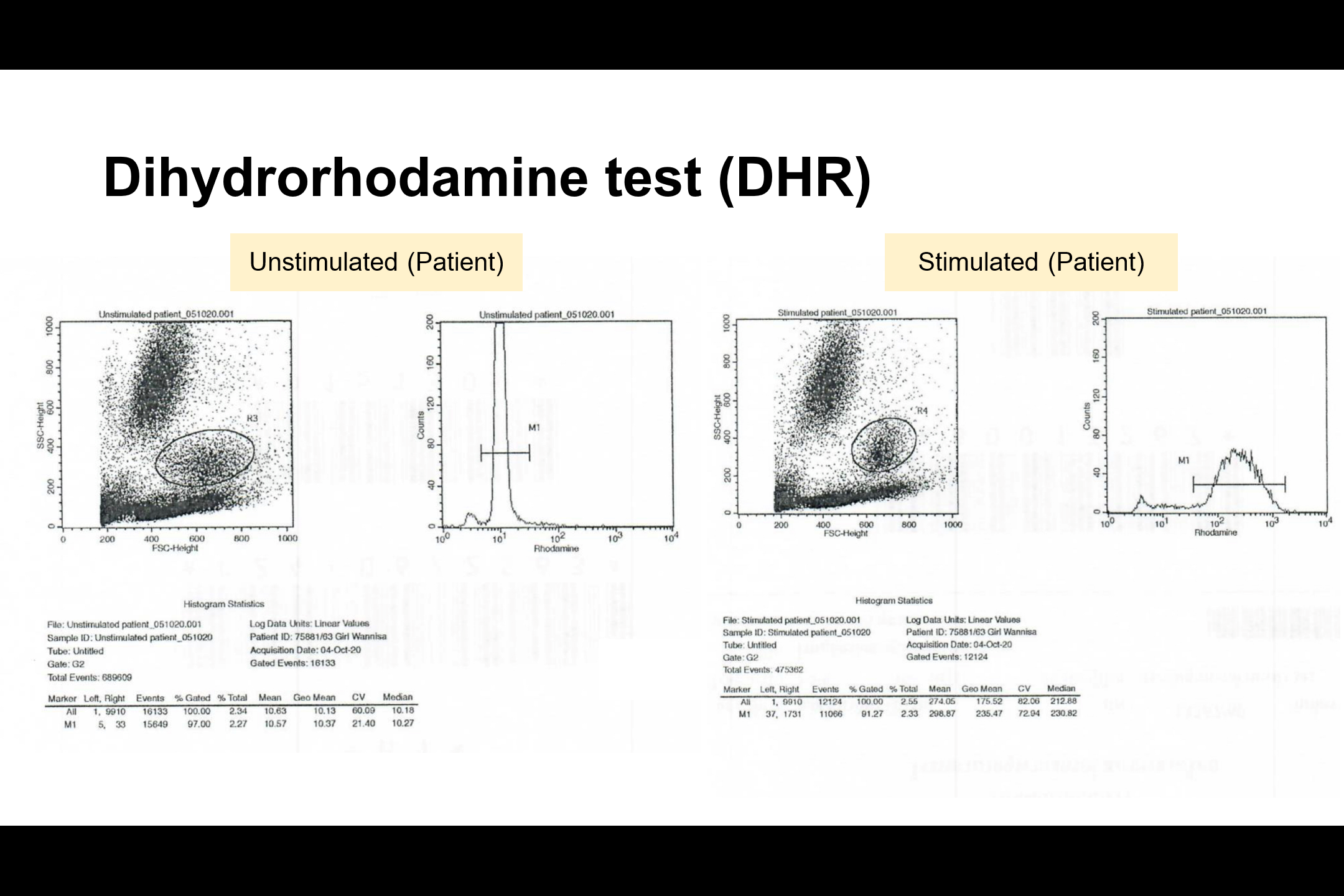


| **CXR** |
| --- |
| Data not available |

| EKG |
| --- |
| Heart rate 90 /min regular  Normal axis  No chamber enlargement by voltage |

| **CT brain**  (with contrast) |
| --- |
| Data unavailable |

| **MRI brain** |
| --- |
| **Data unavailable** |

| MRS brain |
| --- |
| **Data unavailable** |

| PET scan |
| --- |
| **Data not available** |

| Ultrasound whole abdomen  (repeat) |
| --- |
| - Much regression of hepatic microabscess and cystic lesion seen. - Resolution of splenic abscesses |

| **Ultrasound brain** |
| --- |
| **Data not available** |

| **CSF profile** | | | |
| --- | --- | --- | --- |
| Test name | Result | Unit | Reference Value |
| Color  WBC  RBC  Protein  Sugar | Clear  0  0  normal  normal | /mm3  /mm3  mg/dl  mg/dl | Clear  0-5  0-2 |

| CSF Gram stain |
| --- |
| **No organism** |

| **CSF culture** |
| --- |
| **No bacterial growth** |

| **CSF PCR for Herpes virus** |
| --- |
| **Negative** |

| **CSF amino acid** |
| --- |
| **Data not available** |

| **CSF bacterial antigen test** |
| --- |
| **Negative for**  *N. meningitidis serogroup A, C, Y, W-135*  *Streptococcus pneumoniae*  *Haemophilus influenzae type b*  *Group B Streptococcus species,*  *Escherichia coli* |

| **CSF lactate** |
| --- |
| **Data not available** |

| **Nasal Swab for Rapid**  **Influenza A/B/RSV** |
| --- |
| Influenza A Ag: Negative  Influenza B Ag: Negative  RSV Ag: Negative |

| **Endotracheal aspiration**  **Gram stain and Culture** |
| --- |
| Data not available |

| **Stool exam and culture** |
| --- |
| RBC 0-1 cells/HPF  WBC 0-1 cells/HPF  Pasasite not seen  No bacterial growth |

| **Chromosome microarray** |
| --- |
| Data not available |

| **Common mitochondrial DNA mutation**  (MELAS/MERF/Leigh syndrome: A3243G, A8344G, T8993G) |
| --- |
| **Data unavailable** |

Laboratory results – evaluation 3

| **CBC** |
| --- |
| Data not available |

| **Electrolytes** |
| --- |
| Data not available |

| **Blood sugar** | | | |
| --- | --- | --- | --- |
| Test name | Result | Unit | Reference Value |
| Blood sugar | 90 | mg/dL | >60 |

| **Renal function** |
| --- |
| Data not available |

| **Liver function** |
| --- |
| Data not available |

| **Coagulogram** |
| --- |
| Data not available |

| **D-dimer (mg/L)** |
| --- |
| Data not available |

| **Hemoculture** |
| --- |
| Data not available |

| **Serum ammonia** | | | |
| --- | --- | --- | --- |
| Test name | Result | Unit | Reference Value |
| Serum ammonia | Data not available | ug/dl | 30-120 |

| **Blood lactate (venous)** | | | |
| --- | --- | --- | --- |
| Test name | Result | Unit | Reference Value |
| Venous lactate | Data not available | mmol/L | 1.8-2.2 |

| **Urinalysis** |
| --- |
| Data not available |

| **Flow cytometry analysis** | | |
| --- | --- | --- |
|  | **Age 1 year 2 months** | **Normal range** |
| Absolute total lymphocyte (cell/mcl) | 12370 ↑ | 2180-8270 |
| %Total lymphocyte | 69.3 ⟷ | 44-72 |
| Absolute CD3(cell/mcl) | 6680 ↑ | 1460-5440 |
| %CD3 | 54.0 ⟷ | 53-81 |
| Absolute CD4(cell/mcl) | 3340 ⟷ | 1020-3600 |
| %CD4 | 27.0 ↓ | 31-54 |
| Absolute CD8(cell/mcl) | 3093 ↑ | 570-2230 |
| %CD8 | 25 ⟷ | 16-38 |
| Absolute CD19(cell/mcl) | 4206 ↑ | 500-1500 |
| %CD19 | 34 ↑ | 19-31 |
| Absolute CD16+56(cell/mcl) | 1484 ↑ | 59-1178 |
| %CD56 | 12 ⟷ | 3-30 |

| **Immunoglobulin level** | | |
| --- | --- | --- |
|  | **Age 1 year (before IVIG)** | **Normal range** |
| **IgG (mg/dl)** | 1090 ⟷ | 223-1099 |
| **IgM(mg/dl)** | 104 ↑ | 8-100 |
| **IgA(mg/dl)** | 174 ↑ | 1-73 |

| **Dihydrorhodamine test (DHR)** |
| --- |
| Positive |


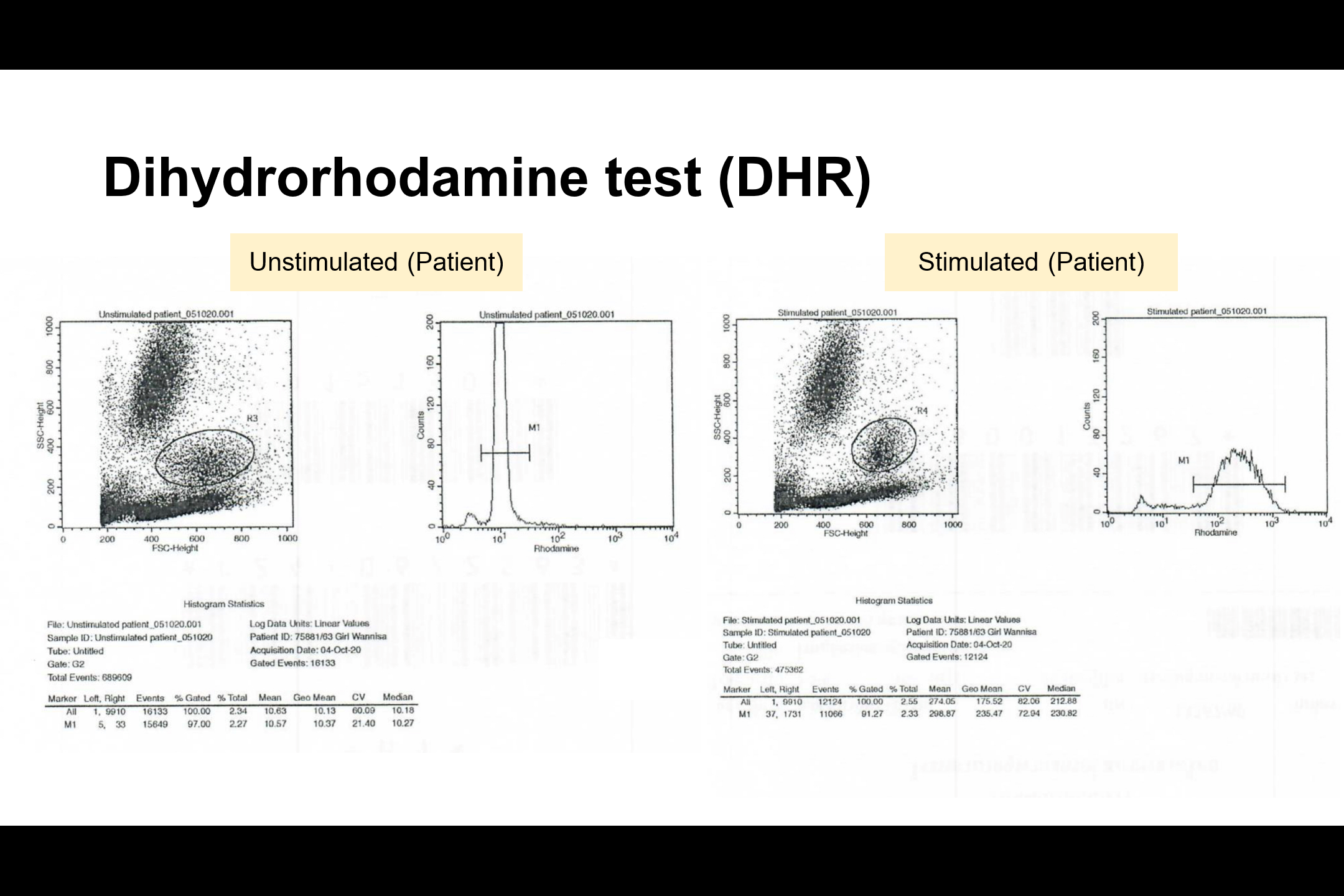


| **CXR** |
| --- |
| Data not available |

| EKG |
| --- |
| Heart rate 90 /min regular  Normal axis  No chamber enlargement by voltage |

| **CT brain**  (with contrast) |
| --- |
| Data unavailable |

| **MRI brain** |
| --- |
| **Data unavailable** |

| MRS brain |
| --- |
| **Data unavailable** |

| PET scan |
| --- |
| **Data not available** |

| Ultrasound whole abdomen  (repeat) |
| --- |
| - Repeat USG whole abdomen at day 37 after total course of antibiotics 5 weeks - Much regression of hepatic microabscess and cystic lesion seen only 3 mm. - Resolution of splenic abscesses |

| **Ultrasound brain** |
| --- |
| **Data not available** |

| **CSF profile** | | | |
| --- | --- | --- | --- |
| Test name | Result | Unit | Reference Value |
| Color  WBC  RBC  Protein  Sugar | Clear  0  0  normal  normal | /mm3  /mm3  mg/dl  mg/dl | Clear  0-5  0-2 |

| CSF Gram stain |
| --- |
| **No organism** |

| **CSF culture** |
| --- |
| **No bacterial growth** |

| **CSF PCR for Herpes virus** |
| --- |
| **Negative** |

| **CSF amino acid** |
| --- |
| **Data not available** |

| **CSF bacterial antigen test** |
| --- |
| **Negative for**  *N. meningitidis serogroup A, C, Y, W-135*  *Streptococcus pneumoniae*  *Haemophilus influenzae type b*  *Group B Streptococcus species,*  *Escherichia coli* |

| **CSF lactate** |
| --- |
| **Data not available** |

| **Nasal Swab for Rapid**  **Influenza A/B/RSV** |
| --- |
| Influenza A Ag: Negative  Influenza B Ag: Negative  RSV Ag: Negative |

| **Endotracheal aspiration**  **Gram stain and Culture** |
| --- |
| Data not available |

| **Stool exam and culture** |
| --- |
| RBC 0-1 cells/HPF  WBC 0-1 cells/HPF  Pasasite not seen  No bacterial growth |

| **Chromosome microarray** |
| --- |
| Data not available |

| **Common mitochondrial DNA mutation**  (MELAS/MERF/Leigh syndrome: A3243G, A8344G, T8993G) |
| --- |
| **Data unavailable** |

Laboratory results - rWES

Diagnostic sheet – evaluation 1

Diagnostic sheet – evaluation 2

Diagnostic sheet – evaluation 3

Doctor order sheet – evaluation 1

Doctor order sheet – evaluation 2

Doctor order sheet – evaluation 3
